# Surprise-related activation in the nucleus accumbens interacts with music-induced pleasantness

**DOI:** 10.1093/scan/nsz019

**Published:** 2019-03-20

**Authors:** Ofir Shany, Neomi Singer, Benjamin Paul Gold, Nori Jacoby, Ricardo Tarrasch, Talma Hendler, Roni Granot

**Affiliations:** 1 Sagol Brain Institute, Tel Aviv Sourasky Medical Center, Tel Aviv, Israel; 2 School of Psychological Sciences, Tel Aviv University, Tel Aviv, Israel; 3 Sagol School of Neuroscience, Tel Aviv University, Tel Aviv, Israel; 4 Montreal Neurological Institute, McGill University, Montreal, QC, Canada; 5 International Laboratory for Brain, Music and Sound Research, Montreal, QC, Canada; 6 The Center for Science and Society, Columbia University, New York, NY, USA; 7 School of Education, Tel Aviv University, Tel Aviv, Israel; 8 Sackler School of Medicine, Tel Aviv University, Tel Aviv, Israel; 9 Musicology Department, Hebrew University of Jerusalem, Jerusalem, Israel

**Keywords:** valence, fMRI, music, nucleus accumbens, surprise

## Abstract

How can music—merely a stream of sounds—be enjoyable for so many people? Recent accounts of this phenomenon are inspired by predictive coding models, hypothesizing that both confirmation and violations of musical expectations associate with the hedonic response to music via recruitment of the mesolimbic system and its connections with the auditory cortex. Here we provide support for this model, by revealing associations of music-induced pleasantness with musical surprises in the activity and connectivity patterns of the nucleus accumbens (NAcc)—a central component of the mesolimbic system. We examined neurobehavioral responses to surprises in three naturalistic musical pieces using fMRI and subjective ratings of valence and arousal. Surprises were associated with changes in reported valence and arousal, as well as with enhanced activations in the auditory cortex, insula and ventral striatum, relative to unsurprising events. Importantly, we found that surprise-related activation in the NAcc was more pronounced among individuals who experienced greater music-induced pleasantness. These participants also exhibited stronger surprise-related NAcc–auditory cortex connectivity during the most pleasant piece, relative to participants who found the music less pleasant. These findings provide a novel demonstration of a direct link between musical surprises, NAcc activation and music-induced pleasantness.

## Introduction

For many people, music is a source of highly pleasing emotional experiences. But how can this abstract stimulus, which does not possess a clear physiological or material benefit, evoke such emotional responses? One neuropsychological mechanism by which music is believed to achieve this impact is musical expectancy—namely the constant establishment of predictions regarding the ‘what’ and ‘when’ of future auditory events and their subsequent fulfillment or violation (Meyer, 1956; Huron, 2006). This mechanism draws a great deal of theoretical and scientific interest, as it emphasizes the role of musically-inherent properties in provoking emotional responses to music. In addition, this possibility resonates with the growingly accepted view of the brain as a predictive machine (Friston, 2005; Barrett, 2017; Koelsch *et al.*, 2018). Here we focus on the theoretical notion that expectancy violations, hereinafter termed musical surprises, are encoded by mesocorticolimbic regions that are usually triggered upon the anticipation or consumption of primary and secondary rewards (Gebauer *et al.*, 2012; Salimpoor *et al.*, 2015).

But how exactly can failed expectations gain a hedonic impact? From a predictive-coding viewpoint, surprises provide novel information that may refine an organism’s prediction of future events (i.e. a ‘prediction error’). Updating of predictions can promote more successful interactions of the organism with its ever-changing environment. Thus, learning about new perceptual features of an unexpected stimulus and determining whether the surprise was better or worse than expected are essential for directing future actions (den Ouden *et al.*, 2012). This process of signaling reward prediction error—the degree to which an incoming stimulus matches the expected level of reward—is supported by the mesolimbic system in the brain (Bromberg-Martin *et al.*, 2010; Schultz, 2010; Berridge and Kringelbach, 2013). Correspondingly, contemporary models of musical affect hypothesize that the continuous cycles of musical expectations and surprises that comprise a musical composition invigorate activation within the same mesocorticolimbic pathway, and thereby give rise to the experience of pleasantness throughout music listening (Vuust and Kringelbach, 2010; Gebauer *et al.*, 2012; Vuust and Witek, 2014; Salimpoor *et al.*, 2015; Koelsch *et al.*, 2018). A major node within this ‘reward system’, the nucleus accumbens (NAcc; which integrates affective, cognitive and motivational information), is believed to play a pivotal role in integrating musical prediction errors with their hedonic impact. Moreover, the functional interactions of this region with cortical areas involved in the processing of acoustic and structural features of music, such as the superior temporal gyrus (STG) and inferior frontal gyrus (IFG), are assumed to underlie the integration of musical expectations with reward sensation (Salimpoor *et al.*, 2013, 2015).

To date, one line of research using neuroimaging, behavioral and physiological measures associated musical surprises with transient increments in emotional arousal (Steinbeis *et al.*, 2006), decreased pleasantness (Steinbeis *et al.*, 2006; Koelsch *et al.*, 2008a, 2008b) and neural activations in regions supporting the processing of perceptual and syntactic-like features of incoming musical stimuli, such as the auditory cortex, IFG and basal ganglia (Maess *et al.*, 2001; Koelsch *et al.*, 2002, 2005; Tillmann *et al.*, 2003, 2006; Koelsch *et al.*, 2008b; Seger *et al.*, 2013). Yet, these studies typically implemented short and artificial musical sequences (cf. Koelsch *et al.*, 2008b) and focused on responses to highly dissonant or syntactically inappropriate chords that terminate a musical passage (Pearce *et al.*, 2010; Miles *et al.*, 2017). While usage of such controlled stimuli allows the examination of responses to violation of specific musical features, it limits the ability to test the association of surprise-related responses with the range of emotions that can be induced by prolonged naturalistic music (Salimpoor *et al.*, 2015).

Other studies have focused on the characterization of neural and physiological responses to highly pleasant music by using naturalistic music, while alluding to the potential role of surprises in music-induced pleasantness somewhat indirectly. These include several neuroimaging studies which associated elevated mesocorticolimbic activation and dopaminergic transmission within its major nodes with pleasant musical experiences (Blood and Zatorre, 2001; Menon and Levitin, 2005; Mitterschiffthaler *et al.*, 2007; Trost *et al.*, 2012). Recently, NAcc activation and its connections with the auditory cortex predicted the size of monetary investments in purchasing unfamiliar music (Salimpoor *et al.*, 2013), thus raising the possibility that implicit musical expectations (i.e. expectations formed through statistical learning of regularities of a particular musical genre or piece) may have governed the observed mesocorticolimbic activations (Salimpoor *et al.*, 2015). However, the association of surprises stemming from the musical structure with reward-related activations was not demonstrated. Finally, another line of studies linked pleasurable ‘chills’ (i.e. the sensation of goosebumps) to unexpected musical material, yet without explicitly modeling or annotating surprise level beforehand (Grewe *et al.*, 2005, 2007; Guhn *et al.*, 2007).

Hence, while musical surprises are theorized to play a pivotal role in shaping the hedonic experience in music, their neural correlates and how these may associate with the overall pleasantness experienced during exposure to naturalistic musical pieces has yet to be tested. More specifically, the processing of musical surprises by the reward system, especially the NAcc, and whether this varies as a function of individual differences in music-induced pleasantness, remains to be demonstrated. Therefore, in the current study we depict neurobehavioral responses to musical surprises as they occur throughout naturalistic musical pieces and test their interaction with music-induced pleasantness. Participants listened in the MRI scanner to three piano pieces in which musical surprises were annotated by an independent group of musically trained individuals. Subsequently, participants reheard the pieces while continuously rating their subjective feelings of pleasantness and arousal. We examined the relationship between surprise processing and individual differences in pleasantness, by capitalizing on the notion that different individuals may experience the same musical pieces as more or less rewarding (Grewe *et al.*, 2007; Guhn *et al.*, 2007; Salimpoor *et al.*, 2011). Based on previous studies, we hypothesized that surprises would elicit changes in the reported emotional experience (hypothesis 1). Specifically, we expected that surprises would associate with enhanced transient arousal (Steinbeis *et al.*, 2006; Koelsch *et al.*, 2008a; Egermann *et al.*, 2013) and with either an increase (Grewe *et al.*, 2005, 2007; Guhn *et al.*, 2007) or decrease (Steinbeis *et al.*, 2006; Koelsch *et al.*, 2008a, 2008b; Egermann *et al.*, 2013) in the reported valence. At the neural level, we expected to replicate and extend previous studies by showing that, under naturalistic listening conditions, moments of expectancy violation would be processed by a ‘cognitive’ frontal and sensorimotor-related network, as well as by mesolimbic regions that encode reward prediction errors such as the NAcc (hypothesis 2). Importantly, we hypothesized that neural activation instigated by musical surprises in the NAcc would interact with the overall level of pleasantness experienced throughout the musical compositions (hypothesis 3). Specifically, we presumed that greater surprise-related activation will be found in the NAcc among listeners who experienced the music as more pleasant relative to both unsurprising (US) events (hypothesis 3a), and to listeners who experienced the music as less pleasant (hypothesis 3b). Finally, we also expected that participants who felt more pleasantness during a certain musical piece would show enhanced coupling of the NAcc with the auditory cortex in response to musical surprises (hypothesis 4).

## Methods

The current paper presents a novel analysis of a data set (Singer *et al.*, 2016) which was previously used to develop a method for highlighting large-scale networks that track the ongoing music-induced emotional experience and to explain how such shared representation is related to a wide set of musical features (including musical surprises). Here we incorporate behavioral measures (affective ratings) and region of interest (ROI) analysis in an event-related fMRI design, in order to test the association between prediction violation in music, NAcc responsiveness and music-induced pleasantness.

### Participants

Forty healthy volunteers (22 females; *M*_age_ = 25.5 ± 3.6 years) participated in the study (see Supplementary methods for additional details). Participants listened to three musical pieces; termed hereafter as Glass, Mussorgsky and Ligeti (see details below). Valid data for the behavioral analysis were available for 39 participants in Ligeti (*M*_age_ = 25.6 ± 3.58; 21 females), 38 participants in Glass (*M*_age_ = 25.72 ± 3.51; 21 females) and 37 participants in Mussorgsky (*M*_age_ = 25.41 ± 3.69; 19 females). Valid data for fMRI analysis were available for 31 participants in Ligeti (*M*_age_ = 25.91 ± 3.71; 17 females), 28 participants in Glass (*M*_age_ = 25.87 ± 3.81; 16 females) and 28 participants in Mussorgsky (*M*_age_ = 25.94 ± 3.75; 16 females), due to technical issues and exaggerated head motions (see criteria in Supplementary methods).

#### Musical stimuli and annotation of musical surprises

The musical stimuli are consisted of three naturalistic piano pieces: ‘The Hours’ by Phillip Glass (light film music; 7:03 min), ‘Night on Bald Mountain’ by Modest Mussorgsky (19th century Russian romantic music; 10:57 min) and ricercatas no. 1 and no. 2 from ‘Musica Ricercata’ by György Ligeti (20th century art music; 0:00–2:57 min and 2:58–7:51 min, respectively).

These three pieces of western art music were selected due to their capability to elicit a range of dynamic emotional experiences (as determined in a pre-test; see Supplementary methods) and musical surprises. More specifically, these pieces were suitable for testing responses to musical surprises for a number of reasons. The Glass and Ligeti pieces are both relatively simple to model in terms of pitch content or harmonic structure. Given the relative simplicity of these pieces, we expected that surprises could be recognized in them quite clearly. Mussorgsky’s ‘Night on Bald Mountain’ is much more complex than the latter two pieces, and thus it could be harder to keep track of this piece—but this piece was selected since it was previously rated as highly surprising (Krumhansl, 1997). Importantly, the prolonged duration of these three pieces and the dynamic nature of their musical structure allowed us to test a sufficient amount of musical events that vary in their surprising degree from each piece and to contrast their effects against those produced by US events from the same musical context. Musical characteristics of the three pieces, which differed widely between pieces, are detailed in the Supplementary methods. Note that we treated Ligeti’s ricercatas as separate pieces and distinguished between two qualitatively different subsections of Mussorgsky’s piece—parts A (0:00–7:32) and B (7:33–10:57)—to account for the effect of musical surprises within a relatively uniform musical context.

Surprising musical events were annotated by 20 musically trained judges who did not participate in the current experiment (*M*_age_ = 26.15 ± 5.04; 8 females; *M*_experience_ = 15.2 ± 5.17). During the surprise-annotation procedure the judges marked the onset of surprising musical events and provided verbal comments regarding the musical features that elicited each surprise. These data were used to identify specific surprising events in each piece. Ranking of surprise degree was defined by adopting a crowd-sourcing approach—summing the number of judges who indicated that a particular musical event was surprising. Eventually, we identified 34 surprises in Glass’s piece (Min_rank_  = 2; Max_rank_ = 19; *M*_rank_ = 6.76 ± 4.67), 56 in part A of Mussorgsky’s piece (Min_rank_ = 2; Max_rank_ = 19; *M*_rank_ = 5.54 ± 3.78), 14 in part B of Mussorgsky’s piece (Min_rank_ = 2; Max_rank_ = 15; *M*_rank_ = 5.5 ± 4.6), 16 in Ligeti’s first ricercata (Min_rank_ = 2; Max_rank_ = 14; *M*_rank_ = 6.93 ± 4.05) and 15 in Ligeti’s second ricercata (Min_rank_ = 2; Max_rank_ = 17; *M*_rank_ = 8.26 ± 4.92). Note that we did not analyze part B of Mussorgsky’s piece and Ligeti’s first ricercata, due to a limited number of valid surprises (see Supplementary methods for inclusion and exclusion criteria for surprises).

The resulting surprise indices were used to define categorically distinct experimental conditions as follows: in Glass and Mussorgsky, the range of surprise indices allowed us to distinguish between highly surprising and low-surprising events based on a median split, hereinafter termed as HS and LS, respectively (Glass: median = 6, *M*_rankHS_ = 10.18 ± 4.33, *M*_rankLS_ = 3.35 ± 1.22; Mussorgsky: median = 5, *M*_rankHS_ = 8.21 ± 3.67, *M*_rankLS_ = 2.85 ± 0.89). Only one level of surprise was defined in Ligeti’s second ricercata due to the small amount of available surprises in this piece. Importantly, we also specified a control condition, consisting of completely US events ranked 0. The US events were randomly selected from beginnings of musical bars. We reasoned that such events would provide a fair control for salience, as they typically coincide with musical events such as strong beats or emphasized notes (London, 2012).

For additional details regarding the surprise annotation procedure, exact onsets and rankings of all defined events and musical and acoustical characteristics of surprises, see Supplementary methodsTable S1 and Supplementary results.

#### Experimental design and procedure

The experimental procedure consisted of an fMRI scanning session which was followed by a behavioral ratings session.

#### fMRI task

During the fMRI session participants were instructed to lay still with their eyes closed and to naturally experience each of the three pieces, which were presented in an order that was semi counter-balanced across participants; Glass and Ligeti were presented either first or last (this order varied between participants), and Mussorgsky was always presented between them. Twenty-three of the participants heard the Mussorgsky piece twice in the scanner. When this piece was played twice, its first presentation was always a passive listening session, and during its second presentation participants continuously rated their emotional experience (see Supplementary methods).

Each musical piece was preceded and followed by a 1 min epoch of silence, and a short chromatic scale was presented 30 s prior to the presentation of the musical piece in order to familiarize the participants with auditory stimulation in the scanner. The musical stimuli were presented at an average sound level of 100 dB using Presentation software (Neurobehavioral Systems, Albany, CA) through MR compatible headphones with active noise cancelation (OptoAcoustics, Israel).

#### Behavioral task

Following scanning, participants reheard the pieces and provided online ratings of their continuous felt emotional experience of valence and arousal using EMujoy softaware (Nagel *et al.*, 2007; see Supplementary methods). The stimuli presentation order in the behavioral session was similar, albeit nor completely identical, to the one that took place in the scanner—i.e. if Glass was presented first and Ligeti was presented last in the scanner (or vice versa), this order remained similar during the behavioral session within participants—but Mussorgsky’s piece was played only once during the behavioral session, as opposed to the fMRI session wherein some participants heard it twice. The continuous rating session was conducted separately from fMRI scanning to avoid influence of the rating task on the neural responses associated with naturalistic listening (Lieberman *et al.*, 2007; Lehne *et al.*, 2013). To further characterize the emotional experience that each piece evoked among participants, they also filled out the Geneva Emotional Musical Questionnaire (GEMS-45; Zentner *et al.*, 2008) and provided subjective reports regarding liking of pieces on a 1–5 Likert scale. A similar scale was used to assess familiarity with pieces.

### Behavioral data analysis

#### Association of surprises with changes in the subjective affective experience

The continuous ratings of valence and arousal were first extracted per subject and down-sampled into a resolution of 1 Hz. To examine how the emotional experience was transiently modulated by musical surprise (hypothesis 1), momentary (i.e. second-by-second) increments or decrements in arousal and valence were indexed as the positive or negative first derivative of the relevant rating, respectively. This resulted in four time series per subject, depicting valence increase, valence decrease, arousal increase or arousal decrease, which were further z-scored within each subject. Next, an event-related affective change index was calculated by separately averaging each of the four valence and arousal changes occurring during 1–4 s following the onset of all surprising or US events (Krumhansl, 1996; Sloboda and Lehmann, 2001; Steinbeis *et al.*, 2006). The event-related affective change indices were then averaged per condition for each subject and submitted to a Friedman’s analysis of variance (ANOVA) in case of the Glass and Mussorgsky pieces. A Wilcoxon matched pairs test was used for comparing between surprising and US events in Ligeti and for post-hoc pairwise comparisons in Glass and Mussorgsky. All comparisons were corrected for multiple comparisons using a false discovery rate (FDR) *P*< 0.05 threshold (Benjamini and Hochberg, 1995).

#### Group clustering based on continuous ratings

To link between neural correlates of surprise processing and individual differences in the overall pleasantness induced by the different pieces, we applied two separate *K*-means clustering analyses on participants’ continuous ratings of valence and arousal. This was obtained by performing a non-hierarchical *K*-means cluster analysis yielding two distinct clusters for each musical piece (using SPSS20 for Windows, IBM, Armonk, New York). Details about the final cluster center values of each group per piece appear in the Supplementary results.

#### fMRI analysis

For fMRI acquisition and data preprocessing details, see Supplementary methods.

#### Statistical analysis

A random-effects general linear model (RFX-GLM) analysis was conducted separately for each musical piece. We defined four regressors in Glass (three experimental conditions and the remaining music), five in Mussorgsky (three experimental conditions and the remaining music in part A and part B) and five in Ligeti (two surprise conditions and the remaining music in ricercata no. 2 and all of ricercata no. 1). All predictors were convolved with a canonical hemodynamic response function. Estimates of the motion correction parameters were added as confound regressors to the model as well. The length of each event was 1 s, and in order to increase the signal-to-noise ratio we assured that events from different conditions were separated by at least 1 TR. Consequently, two and five LS events were omitted from the Glass and Mussorgsky fMRI and behavioral analyses, respectively (Supplementary Table S1).

#### Whole-brain analysis

To detect which brain areas showed sensitivity to degree of surprise (hypothesis 2), we performed a repeated measures ANOVA with surprise level as a within-subjects factor based on the RFX-GLM. We focused on corticostriatal regions that are crucial to our hypothesis by constructing a mask, which was based on results of a prominent meta-analysis on music-evoked emotions (Koelsch, 2014; see Supplementary Figure S1 for additional details). Results of this analysis are reported at a FDR-corrected *P* < 0.05 threshold with a minimal cluster size of 100 contiguous anatomical (1 mm^3^) voxels.

#### ROI analysis

To test our hypothesis that surprise-related activation in the NAcc would interact with individual differences in music-induced subjective pleasantness (hypothesis 3), we conducted an ROI analysis in the NAcc. A 6 mm diameter ROI was defined around coordinates of the right (R) NAcc (Talairach space: *x* = 10, *y* = 6, *z* = 2), driven from a meta-analysis associating this region mostly with rewarding musical experiences (Koelsch, 2014). Beta weights of experimental conditions extracted from the ROI were submitted to a repeated measures ANOVA with surprise level as a within-subjects factor (in Glass and Mussorgsky: HS, LS and US; in Ligeti: surprising and US) and group (high and low pleasantness) as a between-subjects factor. Hypotheses 3a and b, regarding sensitivity to surprise in the high-pleasantness group and group differences in surprise activation, were tested by using *F* contrasts (planned comparisons). We corrected for multiple comparisons by applying an FDR-corrected *P* < 0.05 threshold on all pairwise comparisons across the three pieces (a total of 22 comparisons).

To assess whether the association of surprise with pleasantness is specific to the NAcc and does not extend an auditory processing region, we also tested the group × surprise level interaction in the R STG. A 6 mm diameter functional ROI was defined in the R STG (Talairach space: *x* = 48, *y* = −18, *z* = 8), based on coordinates from Koelsch, 2014 as well. All statistical tests were carried out using Statistica 10 (StatSoft, Tulsa, Oklahoma).

#### Psychophysiological interaction analysis

To test hypothesis 4, regarding an association of pleasantness with surprise-related changes in NAcc–STG connectivity, we conducted a psychophysiological interaction (PPI) analysis (Friston *et al.*, 1997; O’Reilly *et al.*, 2012) while using the above-defined NAcc ROI as a seed region and the above-defined R STG ROI (Salimpoor *et al.*, 2013; Martínez-Molina *et al.*, 2016) as a target region. This RFX-GLM analysis included the two original regressors of the relevant experimental conditions (i.e. surprising and US conditions), a regressor of the physiological variable (i.e. the time course of activity in the seed ROI) and two regressors representing the interaction of the time series of the NAcc with each of the experimental conditions. The analysis was conducted using in-house MATLAB-based software (Gilam *et al.*, 2015). To assess surprise-specific changes in connectivity, we subtracted the PPI estimate of the US condition from that of surprising conditions and tested whether this measure differed between the high- and low-pleasantness groups. A qFDR < 0.05 threshold was applied on all results.

## Results

### 

#### Behavioral results: association of musical surprises with changes in subjective emotionality

**Fig. 1:**
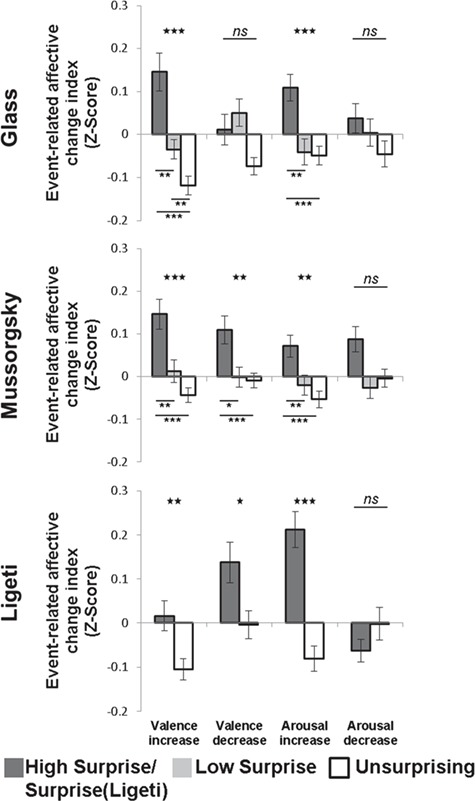
Transient changes in subjective valence and arousal reports are modulated as a function of surprise level. Relative to US events, surprising musical events associated with an increased arousal and valence across all musical pieces (Glass, top; Mussorgsky, center; Ligeti, bottom). In Mussorgsky and Ligeti surprises also related to greater valence decrease. Level of surprise is denoted by grayscale (highest is equal to dark). Stars above the graph bars denote significant main effects in Glass and Mussorgsky and significant pairwise comparisons in Ligeti; asterisks below the graph bars indicate significance of pairwise comparisons in Glass and Mussorgsky; significance of results is indicated: ^*^P <0.05; ^**^P < 0.01; ^***^P ≤ 0.001; qFDR < 0.05. Error bars represent 1 deviation from the mean (SEM).

In accordance with hypothesis 1, we found that surprising events in all three pieces were associated with changes in the reported experience. Specifically, surprises were associated with a greater transient increase in the experienced level of arousal [Glass: *χ*^2^(2) = 19.32, *P* < 0.001; Mussorgsky: *χ*^2^(2) = 12.05, *P* = 0.002; Ligeti: *Z* = 4.45, *P* < 0.001; Figure 1], relative to US events. In Glass and Mussorgsky, post-hoc Wilcoxon’s pairwise comparisons showed that HS associated more robustly with increased arousal, compared to the LS or US conditions (Glass, HS *vs* LS: *Z* = 2.75, *P* = 0.006; HS *vs* US: *Z* = 3.81, *P <* 0.001; Mussorgsky, HS *vs* US: *Z* = 3.23, *P* = 0.001; HS *vs* LS: *Z* = 2.72, *P* = 0.006). Arousal decrease effects were non-significant (NS) in all pieces.

As expected, surprises had both positive and negative effects on valence. Valence increase was modulated by the level of surprise in all three pieces [Glass: *χ*^2^(2) = 16.47, *P* < 0.001; Mussorgsky: *χ*^2^(2) = 14.49, *P* < 0.001; Ligeti: *Z* = 2.60, *P* = 0.009]. Post-hoc comparisons showed that these effects in Glass and Mussorgsky were led chiefly by the HS events, as these were associated with a greater event-related valence increase relative to the LS and US events (Glass, HS *vs* LS: *Z* = 2.65, *P* = 0.008; HS *vs* US: *Z* = 3.75, *P* < 0.001; LS *vs* US: *Z* = 2.97, *P* = 0.003; Mussorgsky, HS *vs* LS: *Z* = 3.04, *P* = 0.002; HS *vs* US: *Z* = 3.87, *P* < 0.001). In addition, we found that valence decrease was also modulated by the surprise level in Mussorgsky’s [*χ*^2^(2) = 10.43; *P* = 0.005] and Ligeti’s (*Z* = 2.44; *P* = 0.01) pieces. In the former piece, HS were associated with a stronger valence decrease relative to US (*Z* = 3.13; *P* = 0.002) and LS (*Z* = 2.15, *P* = 0.031). All results are corrected for multiple comparisons at qFDR < 0.05.

These behavioral results indicate that in the three musical pieces used in this study musical surprises were related to positive changes in the experienced levels of arousal, relative to US events. Effects of surprises on valence were bidirectional, as a surprise-related valence increase was evident in all three pieces, but surprises also related to valence decrease in the Ligeti and Mussorgsky pieces.

#### fMRI results: modulation of fMRI BOLD activity by surprise level

**Fig. 2:**
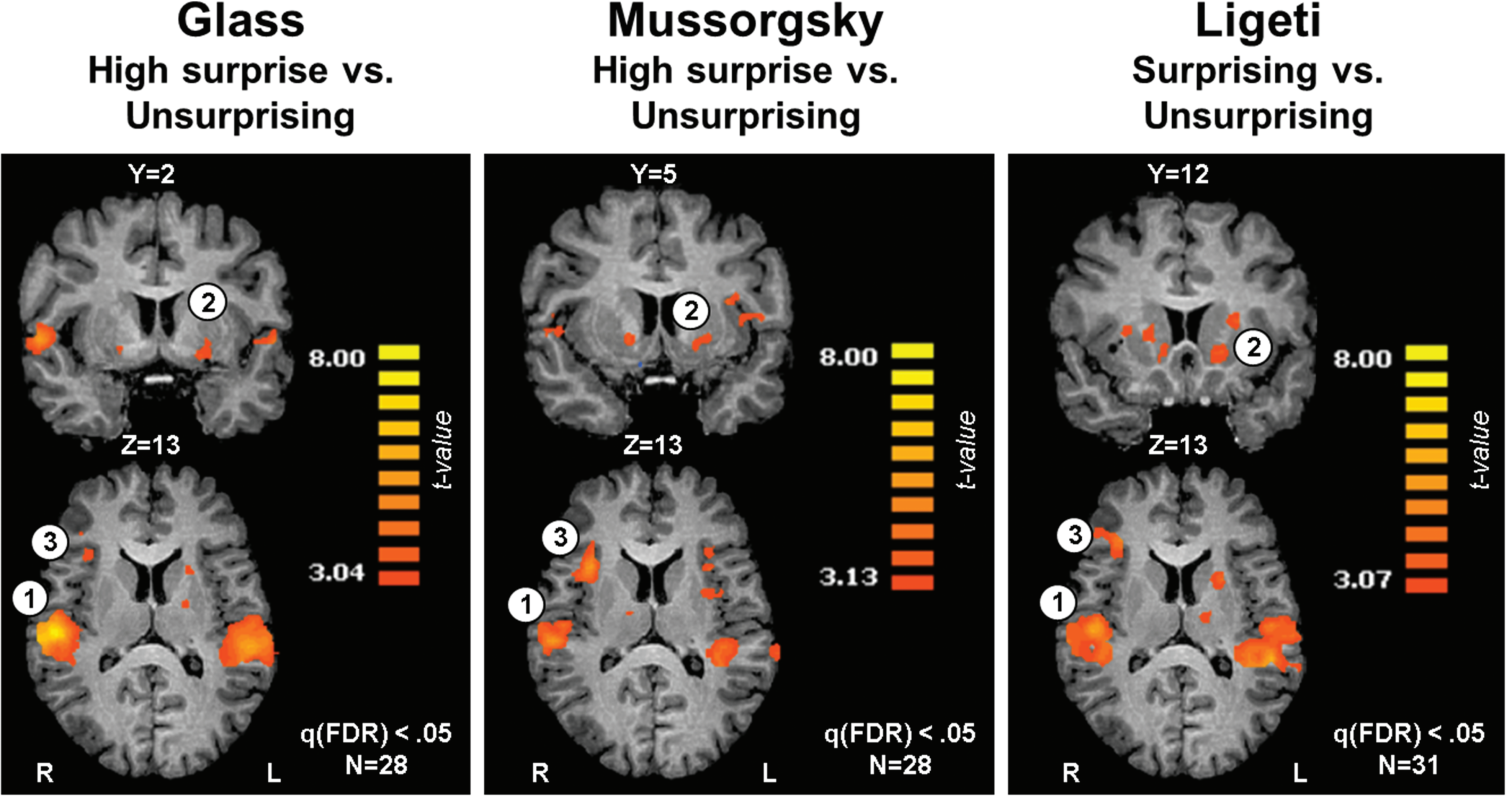
Modulation of brain activation as a function of surprise level. Across three distinct musical pieces [Glass, left (L); Mussorgsky, center; Ligeti, R], surprising events consistently elicited stronger activations relative to US events in the (1) auditory cortex, (2) VS and (3) anterior insula (*P* < 0.05, FDR-corrected). The high surprise *vs* US contrast is depicted for Glass and Mussorgsky. Images are presented in Talairach space and radiological convention.

Supporting hypothesis 2, a main effect of surprise was revealed across pieces in the bilateral STG, different portions of the ventral striatum (VS) and in the R anterior insula (*P* < 0.05, FDR-corrected; Figure 2, note that for Glass and Mussorgsky we present the HS *vs* US contrast). A detailed description of additional clusters showing a main effect of surprise degree and details regarding the direction of surprise-related effects appears in Table 1.

**Table 1:** Modulation of brain activation as a function of surprise level. All regions arising from a random effects ANOVA with surprise level as the within-subject variable, presented per musical piece at a threshold of FDR-corrected *P* < 0.05, using a mask constructed based on results from a recent meta-analysis of music-induced emotions (Koelsch, 2014) with a minimal cluster size of 100 contiguous anatomical (1 mm^3^) voxels. Coordinates of peak activity are given in Talairach space with their *F*-scores and *P*-values. Regions with a minimal cluster size smaller than the defined threshold are presented due to their relevance to the central hypothesis (VS) or recurrence in all pieces (insula) (denoted by ^; see Results). Regions in which main effects of surprise were significant at the whole-brain level (i.e. without applying the mask) are denoted by †. A summary of the specific direction of surprise level effect is presented in the far R column. BA means Broadmann’s area. Anatomical locations were determined using Talairach Daemon (http://www.talairach.org/).

**Brain region**	**BA**	**Side**	***X***	***Y***	***Z***	***F*(2,54)**	***P***	**Voxels (1 mm** ^**8**^ **)**	**Direction of surprise level effect**
**Glass**									
STG†	41, 42 and 22	R	57	−25	10	35.58	0.000	9819	HS > LS > US
Pre-supplementary motor area	6	R	9	14	49	9.81	0.000	796	HS and LS > US
Caudate body		R	12	11	7	7.76	0.001	282	HS > US
Precentral gyrus†	4	R	51	−10	43	13.11	0.000	182	HS > LS > US
Anterior insula^		R	35	20	13	6.61	0.003	80	HS > US
VS/putamen^		R	18	2	−2	6.32	0.003	2	HS > US
STG†	41, 42 and 22	L	−48	−7	1	24.76	0.000	11448	HS > LS > US
Caudate body		L	−18	11	13	7.09	0.002	114	HS > US
VS/putamen		L	−18	−1	−5	7.87	0.001	198	HS and LS > US
		L	−18	12	−2	6.02	0.004	15	HS > US
		L	−21	2	2	5.88	0.005	7	HS > US
		L	−14	14	−5	6.08	0.004	6	HS and LS > US
**Mussorgsky (part A)**									
STG†	41, 42 and 22	R	54	−25	10	23.10	0.000	6564	HS and LS > US
Anterior insula	13	R	36	11	17	10.12	0.000	723	HS and LS > US
VS/putamen^		R	12	2	−2	6.65	0.003	7	HS and LS > US
STG†	41, 42 and 22	L	−48	−19	16	19.75	0.000	4417	HS and LS > US
Middle geniculate† body/thalamus		L	−3	−28	−2	14.77	0.000	1584	HS and LS > US
Anterior insula	13	L	−36	26	10	8.08	0.001	234	HS and LS > US
Middle insula	13	L	−30	2	16	9.64	0.000	174	HS and LS > US
		L	−36	2	8	7.38	0.001	109	HS and LS > US
Anterior STG	22	L	−48	11	1	7.74	0.001	129	HS > LS and US
VS/putamen^		L	−21	5	2	6.80	0.002	8	HS and LS > US
**Ligeti ricercata no. 2**								
STG†	41, 42 and 22	R	45	−13	7	64.94	0.000	7309	S > US
IFG/anterior insula†	46 and 13	R	33	30	13	22.42	0.000	1068	S > US
Caudate body†, extending into VS		R	15	17	7	16.02	0.000	897	S > US
Middle frontal gyrus†	9	R	36	14	28	28.5	0.000	534	S > US
Middle temporal gyrus†	21	R	55	8	−26	37.93	0.000	173	US > S
Claustrum		R	27	14	6	12.70	0.001	140	S > US
STG†	41, 42 and 22	L	−45	−19	4	71.39	0.000	9315	S > US
Dorsal striatum/putamen/		L	−21	8	13	18.57	0.000	390	S > US
VS/putamen		L	−15	12	−2	15.02	0.001	230	S > US
White matter (near putamen)		L	−15	14	−8	11.78	0.002	131	S > US

#### Behavioral results: classification of subgroups experiencing high *vs* low levels of pleasantness in response to the music

**Fig. 3:**
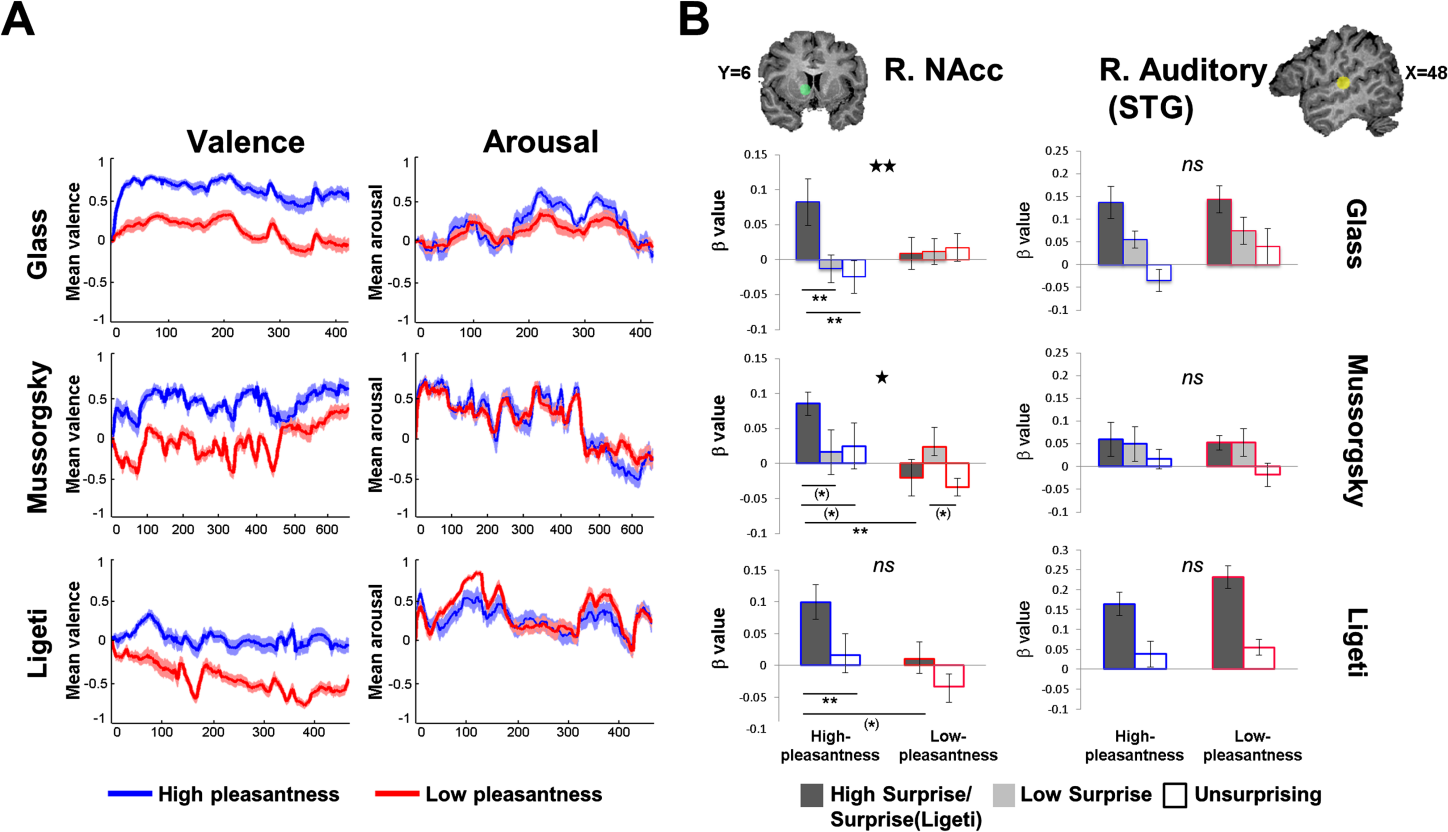
Surprise-related activation and experienced pleasantness interact within the NAcc but not within the STG. **(A)** Subgroups’ clustering based on continuous valence reports. The continuous mean ratings on the scales of valence (L panel) and arousal (R panel) are denoted per group and musical piece (Glass, top; Mussorgsky, center; Ligeti, bottom). Note that while valence ratings clearly differentiate the two groups, arousal ratings do not. Thickness of shading represents 1 SEM. **(B)** ROI analysis in the NAcc and STG. Consistently across musical pieces, the high- but not low-pleasantness groups exhibited stronger activation in response to surprising events in the NAcc, relative to less surprising events (L panel). In contrast, groups showed similar surprise-related activation in the STG (R panel). Level of surprise is denoted by grayscale (highest is equal to dark). The high- and low-pleasantness groups are marked by blue and red contours, respectively. Stars above the graph bars denote interaction effects’ significance: ^*^P < 0.05; ^**^P = 0.01. Asterisks below the graph bars indicate the significance of pairwise comparisons: (*)*P* < 0.05, uncorrected; ^**^P < 0.01; qFDR < 0.05. Error bars represent 1 SEM.

Our *K*-means clustering analysis resulted in the differentiation of two subgroups who reported high- *vs* low-pleasantness per musical piece (Figure 3A; Glass: *N*_high-pleasantness_ = 18/16_fMRI_, *N*_low-pleasantness_ = 20/12_fMRI_; Mussorgsky: *N*_high-pleasantness_ = 15/12_fMRI_, *N*_low-pleasantness_ = 22/14_fMRI_; Ligeti: *N*_high-pleasantness_ = 18/15_fMRI_, *N*_low-pleasantness_ = 21/15_fMRI_; see Supplementary results). Continuous ratings were not available for two participants in Mussorgsky and for one participant in Ligeti, so these participants were omitted from the upcoming fMRI analyses. To note, a similar arousal-based analysis failed to distinguish between participants. Further complementing this classification, liking of musical pieces and additional music-induced positive and negative emotions differed between subgroups (Supplementary Table S2). Importantly, arousal levels were similar in both pleasantness-based subgroups, and the subgroups did not differ in terms of gender, age, musical experience or the distribution of participants who heard Mussorgsky’s piece twice in the scanner (Figure 3A, Supplementary Table S2). However, a slightly higher familiarity with Mussorgsky’s piece was evident in the low-pleasantness group (Wilcoxon’s pairwise comparison: *Z* = −2.10, *P* = 0.041, uncorrected; and *Z* = −2.22, *P* = 0.03, uncorrected, among participants whose fMRI data were analyzed). Hence, the analysis of fMRI data from this piece was repeated with familiarity ratings as a covariate, to exclude the possibility that surprise effects were driven by the familiarity participants had with this piece.

**Fig. 4:**
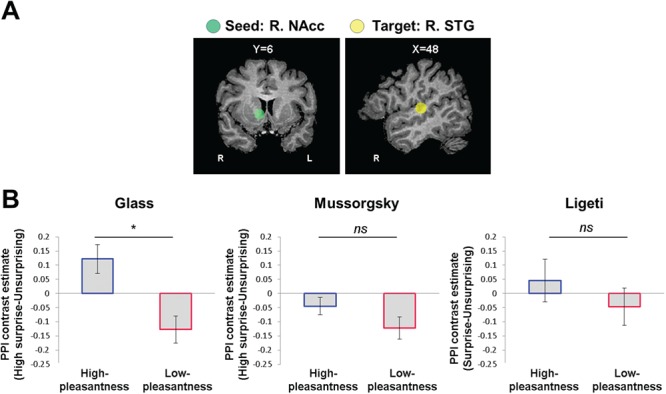
Surprise-related NAcc–STG functional connectivity varies as function of music-induced pleasantness. **(A)** The NAcc (L) and STG (R) ROIs used for a PPI analysis comparing the surprise-related functional connectivity between the high- and low-pleasantness groups. **(B)** Surprise-related connectivity is represented per group and musical piece (Glass, L; Mussorgsky, center; Ligeti, R) as the difference between PPI parameter estimates (beta) of surprising relative to US conditions. High- and low-pleasantness groups are marked by blue and red contours, respectively. The asterisk above the graph bars indicates the significance of group comparisons: ^*^P < 0.005; qFDR < 0.05. Error bars represent 1 SEM.

#### fMRI results: music-induced pleasantness interacts with surprise processing in the NAcc but not in the auditory cortex

In our central analysis we tested the interaction of pleasantness with surprise level in the NAcc ROI. The ANOVA first revealed main effects of surprise level and group which varied across pieces: a main effect of surprise level was evident in Ligeti [*F*(1,28) = 9.67; *P* = 0.004; *ηp^2^* = 0.26] and Glass [*F*(2,52) = 3.69; *P* = 0.031; *ηp^2^* = 0.12] but not in Mussorgsky [*F*(2,48) = 1.65; *P* = 0.204; *ηp^2^* = 0.06], and NS trends for group effects were found in Ligeti [*F*(1,28) = 3.97; *P* = 0.056; *ηp^2^* = 0.12] and Mussorgsky [*F*(1,24) = 3.32; *P* = 0.08; *ηp^2^* = 0.12]—both pointing to greater R NAcc activation in the high-pleasantness group—yet no such trend was present in Glass [*F*(1,26) = 0.007; *P* = 0.93; *ηp^2^* = 0.00].

Conforming our third and main hypothesis, a statistically significant group × surprise level interaction was evident in Glass [*F*(2,52) = 4.69; *P* = 0.013; *ηp^2^* = 0.15] and Mussorgsky [*F*(2,48) = 3.74; *P* = 0.03; *ηp^2^* = 0.13] but not in Ligeti [*F*(1,28) = 0.97; *P* = 0.33; *ηp^2^* = 0.03]. In consistence with hypothesis 3a, according to which the high-pleasantness group will show stronger surprise-related activation in the NAcc, planned comparisons showed that the high-pleasantness group exhibited significantly enhanced activation in response to HS (Glass) and surprising (Ligeti) events, relative to less surprising events [Figure 3B; Glass, HS *vs* LS: *F*(1,26) = 12.09; *P* = 0.001; HS *vs* US: *F*(1,26) = 12.80, *P* = 0.001; LS *vs* US: *F*(1,26) = 0.28, *P* = 0.59; Ligeti, surprising *vs* US: *F*(1,28) = 8.39, *P* = 0.007]. A similar trend was found in Mussorgsky, but significance level of results did not meet the correction for multiple comparisons [HS *vs* LS: *F*(1,24) = 4.11, *P* = 0.05, qFDR < 0.15; HS *vs* US: *F*(1,24) = 4.70, *P* = 0.04, qFDR < 0.15; LS *vs* US: *F*(1,24) = 0.07, *P* = 0.78]. To confirm that surprise-related effects were specific for the high-pleasantness groups, we conducted similar *F* contrasts in the low-pleasantness group. No significant differences were found between surprise conditions for either Glass (HS *vs* US: *P* = 0.81; HS *vs* LS: *P* = 0.93; LS *vs* US: *P* = 0.83), Ligeti (*P* = 0.14) or Mussorgsky (HS *vs* US: *P* = *0*.61; HS *vs* LS: *P* = 0.17; however, an uncorrected trend was found for LS *vs* US: *P* = 0.043, qFDR > 0.1).

Hypothesis 3b regarding group differences in surprise-related activations in the NAcc was partially supported. Significant differences between groups for HS were found in Mussorgsky [HS: *F*(1,24) = 10.19, *P* = 0.004, Hedge’s *G* = 1.21; LS: *P* = 0.87, Hedge’s *G* = 0.026], as well as for surprises at an uncorrected significance level in Ligeti [F(1,28) = 5.00, *P* = 0.033, Hedge’s *G* = 0.84]—both in the expected direction (i.e. high pleasantness > low-pleasantness). In Glass, NAcc activation did not differ between groups for both surprise conditions (HS: *P* = 0.11, Hedge’s *G* = 0.60; LS: *P* = 0.39, Hedge’s *G* = −0.32). *F* contrasts testing group differences in response to the US conditions did not yield significant results in any of the pieces (Glass: *P* = 0.22; Mussorgsky: *P* = 0.1; Ligeti: *P* = 0.26). All pairwise comparisons are corrected at a qFDR < 0.05 threshold.

To sum, HS (surprises in Ligeti) induced higher NAcc activation in the high-pleasantness group in all three pieces, relative to US (trend in Mussorgsky); NAcc activation in response to HS (Mussorgsky) and surprises (Ligeti, trend) was stronger in the high-pleasantness group as compared to that found in the low-pleasantness group. To account for the possible effect of familiarity in Mussorgsky’s piece, we conducted the ANOVA again with familiarity level as a covariate and found that the pattern of results remained similar (see Supplementary results).

To verify that the observed groups effects were specific to the NAcc (Salimpoor *et al.*, 2013; Martínez-Molina *et al.*, 2016), we tested the group × surprise level interaction in the R STG ROI as well. The interaction was NS in either Glass’s (*P* = 0.29), Mussorgsky’s (*P* = 0.64) or Ligeti’s (*P* = 0.27) pieces (Figure 3B).

#### fMRI results: association of music-induced pleasantness with surprise-related NAcc–STG functional connectivity

Finally, we turned to examine how the NAcc–STG coupling in response to surprising *vs* US events interacts with the overall experienced pleasantness. In Glass and Mussorgsky we focused on the connectivity difference between HS and US conditions, as HS rather than LS were consistently linked to stronger neural activations and affective changes relative to US. Partially supporting our fourth hypothesis, we found that surprise level associated with group differences in NAcc–STG coupling in Glass’s piece [high-pleasantness *vs* low-pleasantness: *t*(25) = 3.38, *P* = 0.002, Hedge’s *G* = 1.28, qFDR <0.05; Figure 4B]. Group differences were NS in Mussorgsky [*t*(24) = 1.46, *P* = 0.157, Hedge’s *G* = 0.56] and in Ligeti [*t*(28) = 0.89, *P* = 0.377, Hedge’s *G* = 0.32]. Note that one participant was removed from the analysis in Glass, due to abnormal beta values [>3 SDs from the mean; the *t*-test for group differences in Glass is also significant if this participant is included in the analysis, *t*(26) = 3.01, *P* < 0.006, Hedge’s *G* = 1.1, qFDR < 0.05]. Interestingly, whole-brain functional connectivity maps show that the group differences in surprise-related connectivity from the NAcc are relatively specific to the auditory cortex in Glass and Mussorgsky (Supplementary Figure S2; Glass was analyzed without the outlier participant mentioned above). Moreover, an exploratory analysis revealed that the high-pleasantness group showed a stronger surprise-related connectivity of the NAcc with the ventromedial prefrontal cortex (vmPFC), relative to the low-pleasantness group, in Glass’s piece (*P* < 0.005, cluster-level corrected with 5000 iterations of Monte Carlo simulations; this was the only cluster that met this statistical threshold; see Supplementary Figure S2). The vmPFC plays a critical role in reward valuation (Liu *et al.*, 2011; Bartra *et al.*, 2013), and its connectivity strength with the auditory cortex was found to predict the rewarding value of unfamiliar music (Salimpoor *et al.*, 2013).

## Discussion

In the current study we addressed the theoretical claim that the processing of naturalistic musical surprises and its association with pleasantness is correlated with the functionality of the mesocorticolimbic system—specifically with the activity of reward-related brain regions and their transient connectivity with auditory regions (Gebauer *et al.*, 2012; Salimpoor *et al.*, 2015). In three naturalistic musical pieces, we found that, relative to US events, musical surprises associated with transient changes in the subjective experience—namely with positive changes in reported arousal and with both increases and decreases in reported pleasantness. At the neural level, musical surprises compared to US events activated the auditory cortices, anterior insula and VS. Furthermore, we found that pleasantness and surprises interact in a reward-related brain structure, as subgroups of participants who felt more pleasantness during listening exhibited stronger surprise-related activation in the NAcc. Moreover, this subgroup also showed enhanced surprise-related NAcc–auditory cortex connectivity relative to the low-pleasantness group in the most pleasant piece.

### 

#### Naturalistic musical surprises relate to transient changes in reported pleasantness and arousal

In accordance with theories stressing the role of musical expectations in emotion elicitation (Meyer, 1956; Huron, 2006; Juslin and Västfjäll, 2008), we found that musical surprises related to transient enhancements in arousal and to both positive and negative shifts in valence (Figure 1).

Elevated arousal is repeatedly linked to musical surprises in studies using both artificial (Steinbeis *et al.*, 2006) and naturalistic (Koelsch *et al.*, 2008b; Egermann *et al.*, 2013) musical stimuli. This is consistent with the idea that unexpected events indicate salience, and thereby serve as alerting or reorienting signals that may orchestrate shifts in the organism’s allocation of attentional and metabolic resources—a process tightly linked to heightened arousal (Bradley, 2009; den Ouden *et al.*, 2012).

In contrast to the consistent positive effect on arousal, to date studies found that musical surprises had both positive (Grewe *et al.*, 2005, 2007; Guhn *et al.*, 2007) and negative (Steinbeis *et al.*, 2006; Koelsch *et al.*, 2008a, 2008b; Egermann *et al.*, 2013) effects on transient pleasantness, perhaps due to large differences in the musical stimuli. The surprises identified in the current study were associated with changes in the affective experience of valence, either enhancing or decreasing it, relative to less surprising events. This finding is congruent with the idea that unexpected events *per se* are infused with hedonic value. Such value may stem from an assessment of whether outcomes were better or worse than expected (Shepperd and McNulty, 2002; den Ouden *et al.*, 2012), and also by the ability to integrate the surprising event with previous information (Gebauer *et al.*, 2012).

#### Musical surprises activate cognitive, affective and perceptual regions

Even though the musical pieces differed in their structure and style, activations in the STG, anterior insula and VS were consistently modulated by surprise level across pieces (Figure 2). The STG houses the primary and secondary auditory cortices. These regions match incoming auditory stimuli with pre-existing templates, thereby providing an auditory working memory infrastructure for expectancy generation throughout music listening (Koelsch, 2011; Salimpoor *et al.*, 2015; Trainor and Zatorre, 2015). The anterior insula has a key role in detecting salient stimuli and facilitating attention allocation toward those stimuli by interacting with other brain networks (Menon and Uddin, 2010). Its dorsal portion, which was activated here, is involved in a wide set of emotional and cognitive processes (Kurth *et al.*, 2010), including error monitoring (Chang *et al.*, 2013), reward (Liu *et al.*, 2011; Bartra *et al.*, 2013), musical working memory (Koelsch, 2011; Schulze *et al.*, 2011) and aesthetic appraisals (Brown *et al.*, 2011)—functions which are consistent with the predictive coding framework of musical processing.

The portions of the VS found active here, comprising of the ventral putamen and ventromedial caudate, are known for their involvement in instigating reorientation reactions in response to salient unexpected events, as well as in assigning positive and negative valence to surprises (den Ouden *et al.*, 2009; Haber and Knutson, 2010; den Ouden *et al.*, 2010). Importantly, dopaminergic transmission during pleasurable responses to music was demonstrated in these regions (Blood and Zatorre, 2001; Salimpoor *et al.*, 2011). Taken together, we suggest that the STG, anterior insula and VS activations might support three important processes occurring in response to dynamically forming surprises: monitoring auditory probabilities (STG); facilitating orientation of cognitive and affective resources toward salient musical stimuli (anterior insula); and integrating surprise processing with motivational aspects, possibly resulting in changes in experienced arousal and valence (VS). Further research is needed to untangle these subprocesses from the overall pleasantness.

#### Interaction of music-induced pleasantness and surprise level in NAcc’s activation and functional coupling with the STG

Taking advantage of the natural variability in how music makes different listeners feel, we could demonstrate that there is a link between the level of music-induced pleasantness and surprise-related activity and connectivity of the R NAcc.

Previous work has established that NAcc activation is consistently involved in rewarding musical experiences (Menon and Levitin, 2005; Koelsch *et al.*, 2006; Salimpoor *et al.*, 2011; Trost *et al.*, 2012; Salimpoor *et al.*, 2013). However, these studies did not directly test the hypothesized association of prediction violations with the hedonic experience. Additionally, some of these studies utilized music selected by the participants (Blood and Zatorre, 2001; Salimpoor *et al.*, 2011), thus making it difficult to determine whether reward activity was provoked by novelty of the musical structure or rather by expectations regarding well-known pleasing musical passages or extra-musical associations such as autobiographical memories (Granot, 2017). Our results add to this line of research by indicating that NAcc activation in response to subjectively pleasant and relatively unfamiliar music is related to specific musical surprises stemming from the musical structure and/or from performance cues. This supports the idea that implicit expectations formed by means of statistical learning of regularities throughout one’s exposure to a musical culture or a certain piece (Bharucha, 1994; Huron, 2006) may play an important role in the reward-related response to music (Salimpoor *et al.*, 2013).

While the high- and low-pleasantness subgroups showed differences in surprise-related activations in the NAcc, STG activation was fairly similar between groups (Figure 3B). This suggests that the groups did not differ in terms of perceptual processing and supports the idea that perceptual and emotional processing of music involve distinct neural mechanisms mediated by cortical and subcortical regions, respectively (Peretz, 2010). More broadly, this finding is in agreement with predictive coding frameworks stressing that novel stimuli can deliver both sensory and motivationally valenced prediction error signals, which serve different functions and are processed by sensory-relevant cortices and mesostriatal systems, respectively (den Ouden *et al.*, 2012). Mesocorticolimbic interactions, specifically between the NAcc and STG, are theorized to support the attachment of hedonic value to novel musical patterns (Salimpoor *et al.*, 2015). Indeed, an increase in this functional connection was linked to attachment of reward value to previously unheard music (Salimpoor *et al.*, 2013), and a downregulation of its strength was recently found in participants with specific musical anhedonia (Martínez-Molina *et al.*, 2016). Respectively, we found significantly greater surprise-related STG-NAcc functional connectivity in the high-pleasantness group in the most pleasant composition (by Glass; see Figure 4B and Supplementary results). Together, these findings suggest that surprises are points in which focal differences that emerge in NAcc–STG connectivity are important correlates of the hedonic reaction to music.

### Limitations

Several limitations of this study should be taken into account. First, we used a small number of musical pieces that are identified with the western 19th and 20th art music, which limits the ability to generalize our findings to other musical contexts and genres. The associations reported here should be characterized in additional exemplars from different genres. Second, while surprises were annotated by musicians, surprise-related brain activations were derived from a separate group comprising of both musically trained and non-experienced listeners, who were not asked to report on their subjective experience of surprise. Thus, it remains unclear whether such subjective level of surprise actually corresponded with the surprising degree of the experimental events, nor how individual differences in experience of surprise affected brain activity. Future studies could apply subjective continuous measurements of surprise and/or computational modeling of these events based on musical-structural elements (e.g. Pearce *et al.*, 2010; Egermann *et al.*, 2013). Third, the continuous affective ratings were collected during a repeated listening session, meaning that surprise-related affective changes were obtained when events were less unpredictable. Thus, even though minimal, the influence of explicit familiarity on the affective ratings remains unclear. Fourth, even though the high- *vs* low-pleasantness groups did not differ in terms of age, gender or musical experience (Supplementary Table S2), the assignment of participants to these groups was not random. Therefore, there may be additional between-group differences that can account for the results.

### Conclusions

In this study we examined neurobehavioral responses to musical surprises occurring in naturalistic pieces and their association with listeners’ pleasantness. Our results suggest that surprises constitute auditory ‘alerting signals’ with inherent salience (Bromberg-Martin *et al.*, 2010) that can engage relevant sensory regions and raise arousal levels—as indicated here by the surprise-related arousal effects and activations in the auditory and insular cortices, respectively. Alongside, while processing perceptual features and general salience of musical ‘errors’ may be similar between individuals, processing motivational aspects of surprises by reward centers may vary as a function of the subjective experience of positive valence, as indicated here by the heightened NAcc response to surprises among those who found the music more pleasant. These findings provide evidence for the hypothesized link between naturalistic surprises, affect-related brain activation and attachment of subjective hedonic value to music (Gebauer *et al.*, 2012; Salimpoor *et al.*, 2015).

## Funding

This work has received funding from the European Union's Seventh Framework Program for research technological development and demonstration under grant agreement no. 602186, from the Israeli Centers for Research Excellence Program (I-CORE) on behalf of the Planning and Budgeting Committee and The Israel Science Foundation under grant agreement no. 51/11 (to T.H), and from the Ministry of Science & Technology, Israel & the LE FONDS DE RECHERCHE DU QUÉBEC - NATURE ET TECHNOLOGIES (FRQNT) and LE FONDS DE RECHERCHE DU QUÉBEC - SANTÉ (FRQS) (Grant no. 3-14011, *Music to my brain*, to T.H). The work was additionally supported by the Converging Technologies Scholarship on behalf of the Council for Higher education and the Levie-Edersheim-Gitter Institute for Functional Brain Mapping scholarship (to N.S.).

## 

Conflict of interest None declared.

## Supplementary Material

Suppl.ForSCAN_nsz019
