# Supplementary material for: Surprise-related activation in the nucleus accumbens interacts with music-induced pleasantness
Source: Soc Cogn Affect Neurosci. 2019 Mar 20;14(4):459–70. doi: 10.1093/scan/nsz019 (PMC6523415; doi:10.1093/scan/nsz019)
Supplement: Suppl.ForSCAN_nsz019 [file suppl.forscan_nsz019.docx]

**Supplementary information**

**Contents**

**Supplemental data**

***Figures***

**Figure S1:** Anatomical mask used for whole-brain analysis

**Figure S2:** Group differences in surprise-related NAcc functional connectivity

**Figure S3:** Timeline of experimental events

***Tables***

**Table S1:** Details of experimental events

**Table S2:** Characteristics of high- vs low-pleasantness groups for each musical piece

**Supplemental Methods**

**Supplemental Results**

**Supplemental References**

**Supplemental data**


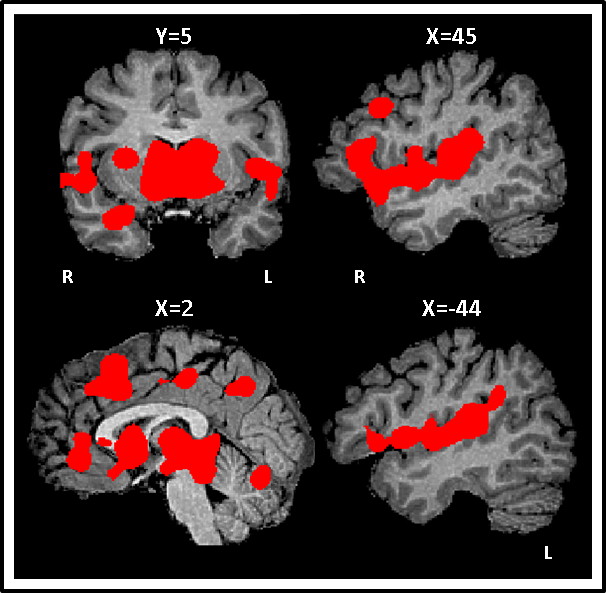


**Figure S1. Anatomical mask used for whole-brain analysis.** For the whole-brain analysis, we used a mask covering regions that are involved in music perception and musical emotions. The mask was driven from a recent meta-analysis on the neural correlates of music-evoked emotions (Koelsch, 2014). Note that in order to construct the mask we applied a statistical threshold of *p*<.05, uncorrected, with a minimum cluster size of 300mm³ on the map produced in that meta-analysis, which is less stringent than the p<.01 (FDR-corrected, with a minimum cluster size of 200 mm³) that is presented in the meta-analysis. We used this threshold since we wanted to cover more brain areas – especially the IFG, Insula and larger portions of the STG - whose activation was previously linked to processing of musical surprises (e.g., Koelsch et al., 2002; Tillmann et al., 2003).


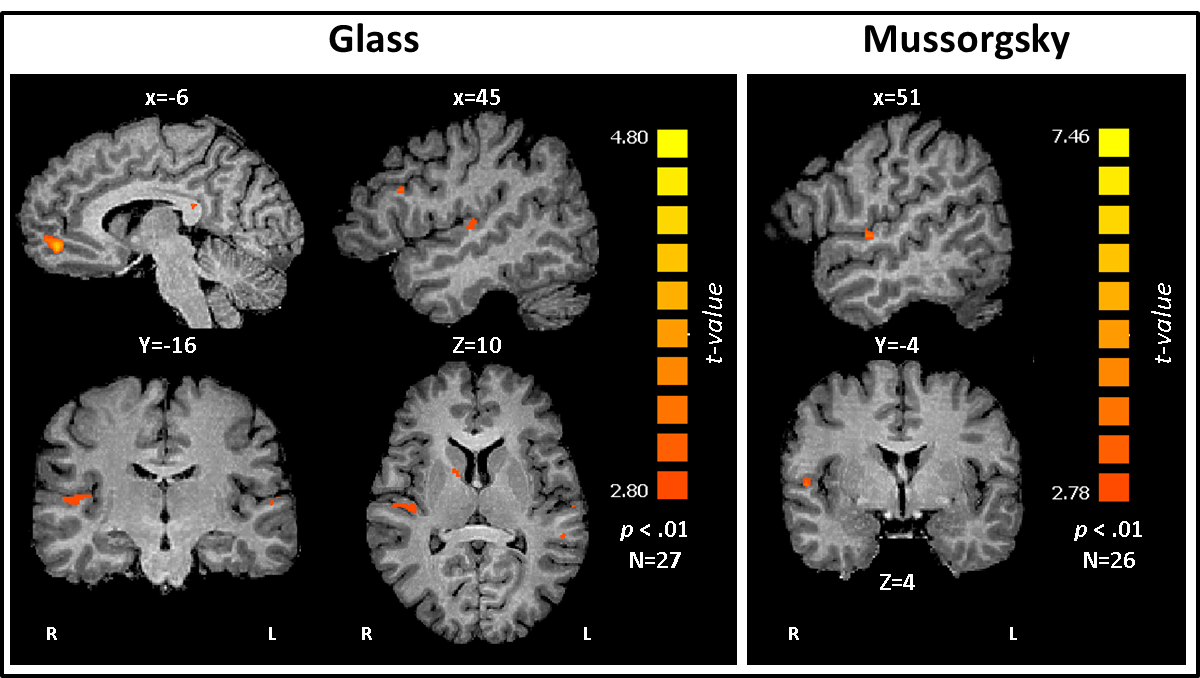


**Figure S2. Group differences in surprise-related NAcc functional connectivity.** The maps present a between-group t-test (i.e. high- vs. low- pleasantness) of the beta estimates for the surprise-related (i.e. high-surprise vs. unsurprising contrast) connectivity from the NAcc seed in both Glass's (left panel) and Mussorgsky's (right panel) pieces. Notice that in both pieces the enhanced functional coupling found in the high- relative to the low-pleasantness group is relatively restricted to the superior temporal gyrus. Interestingly, the high-pleasantness group showed a stronger surprise-related connectivity of the NAcc with the ventromedial prefrontal cortex (vmPFC), relative to the low-pleasantness group, in Glass's piece (significant at *p*<.005, cluster-level corrected with 5000 iterations of Monte Carlo simulations; this was the only cluster that met this statistical threshold). The maps are presented at a lenient threshold of *p*<0.01, uncorrected, using the mask presented in Figure S1.

**
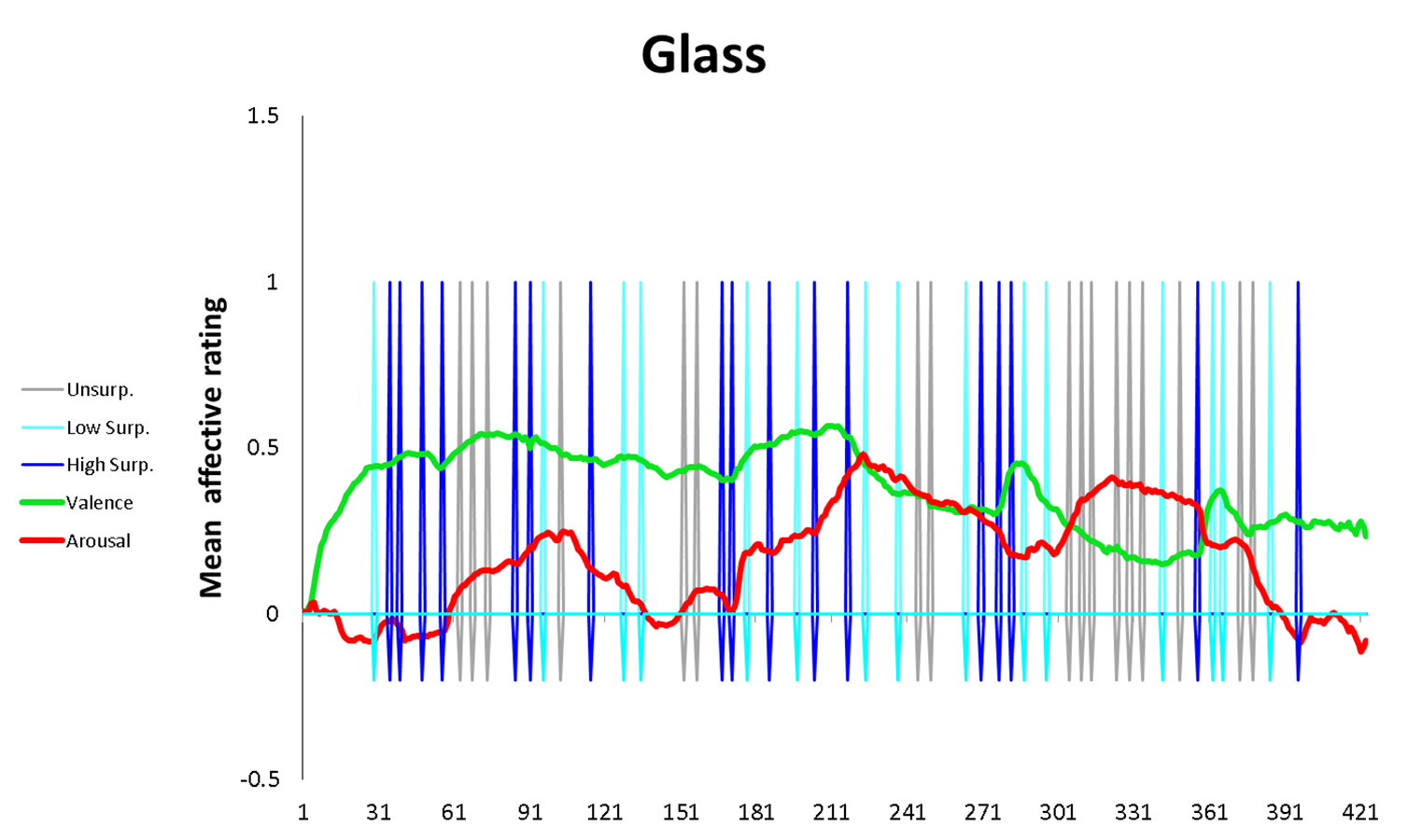

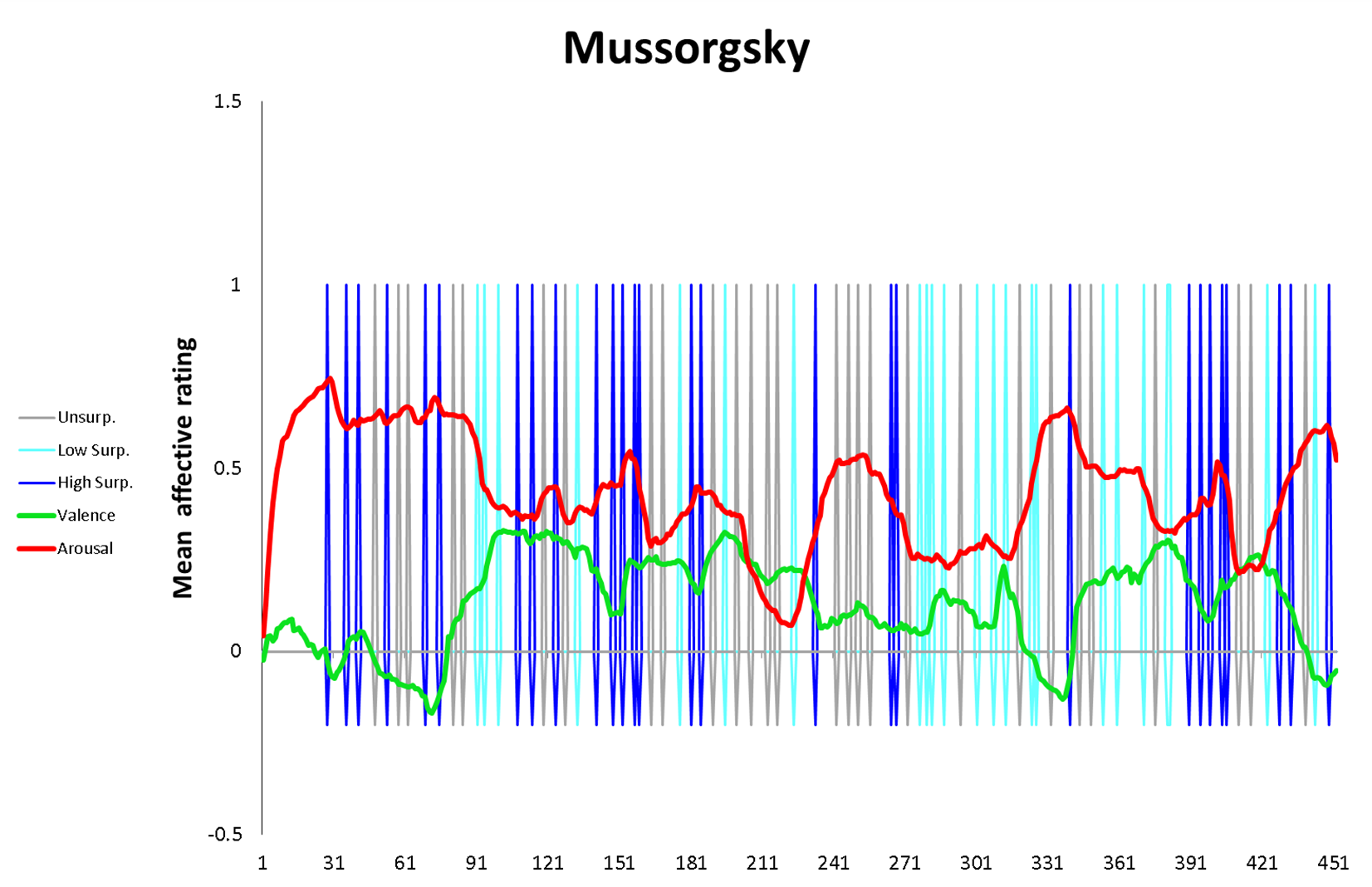
**

**Figure S3:** **Timeline of experimental events**. The figure shows the distribution of surprising and unsurprising events throughout each musical piece (Glass, top; Mussorgsky, center; Ligeti, bottom). Occurrence of surprising and unsurprising events is marked by sticks, whose height and width are arbitrary (dark blue for high surprises in Glass and Mussorgsky and surprises in Ligeti; light blue for low surprises; grey for unsurprising events). The grand mean of the continuous ratings of valence (light green) and arousal (red) are overlaid on the plot of each piece as well. The Y axis denotes the mean of each of these affective ratings, and the X axis denotes time in seconds. Abbreviations stand for: surprise (surp.), seconds (sec).


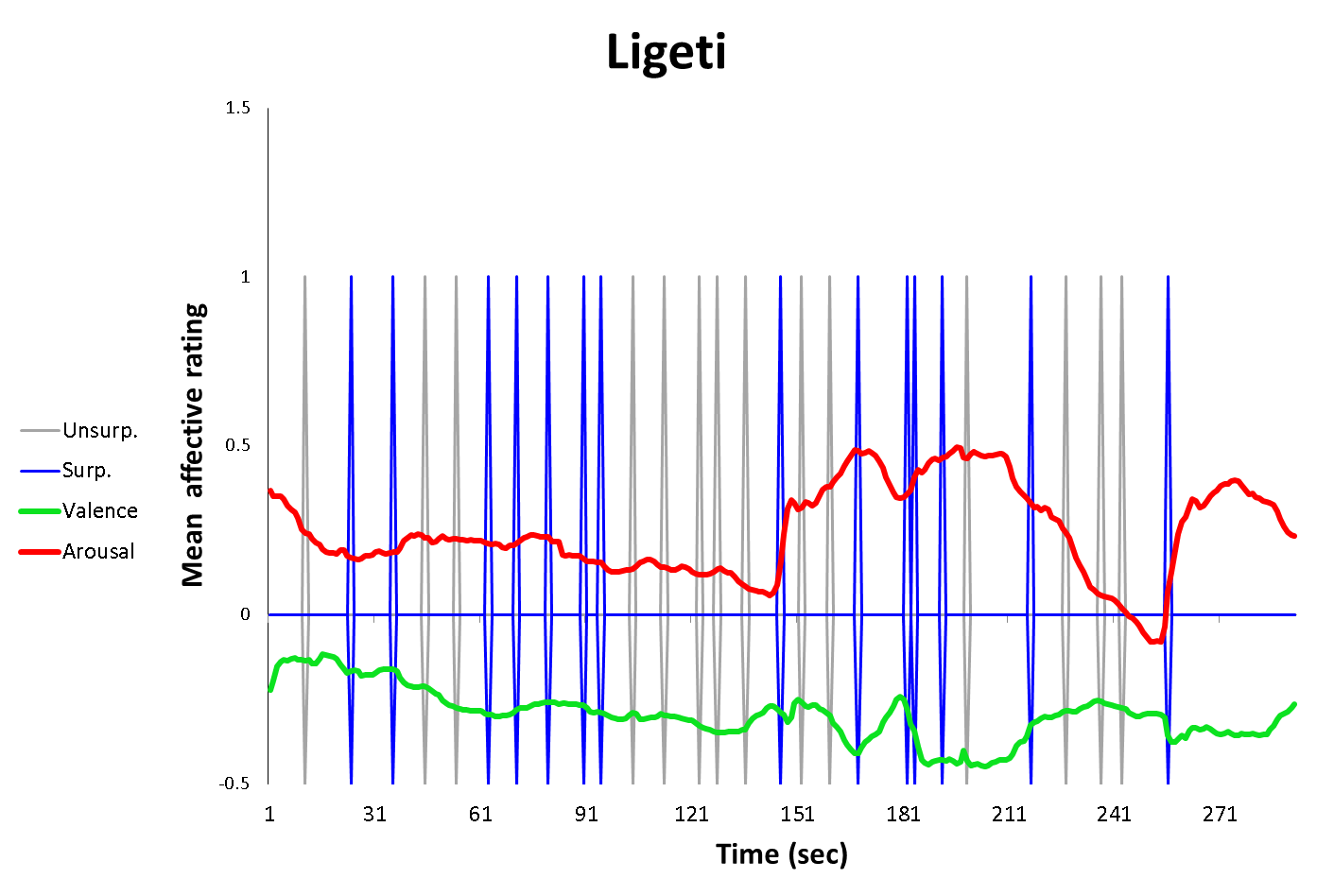


**Table S1. Details of experimental events**


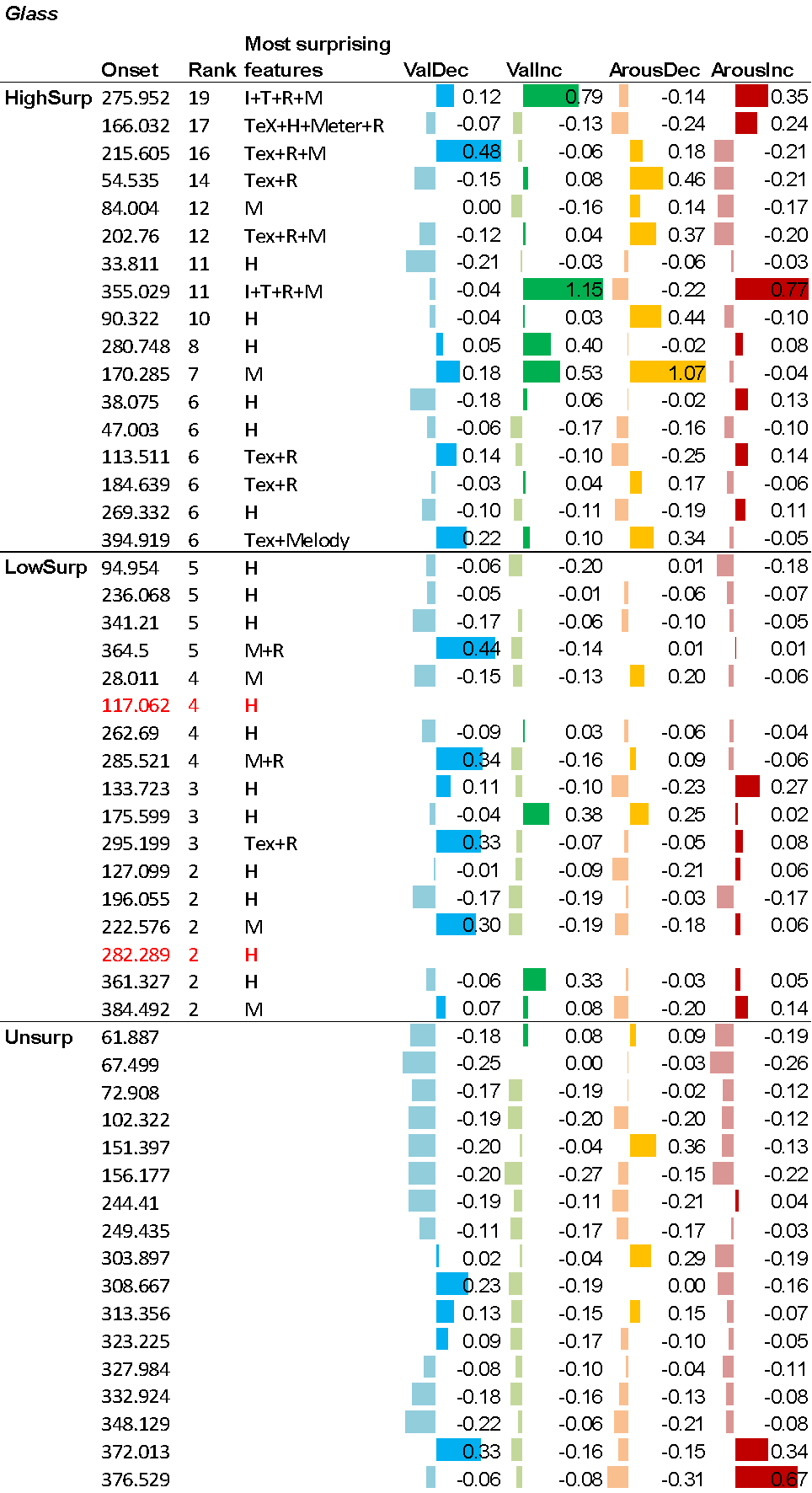


**Table S1.** **Cont.**


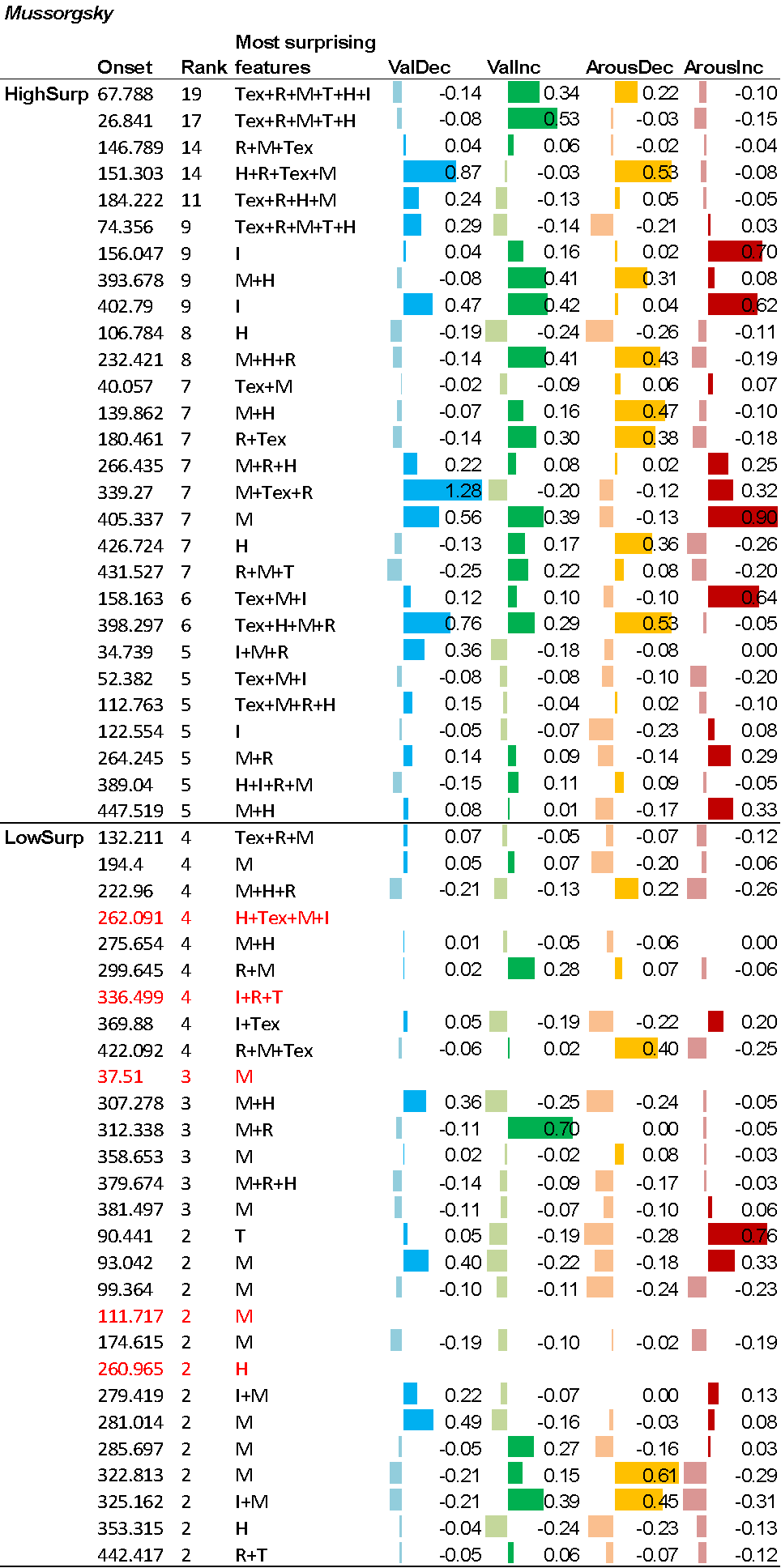


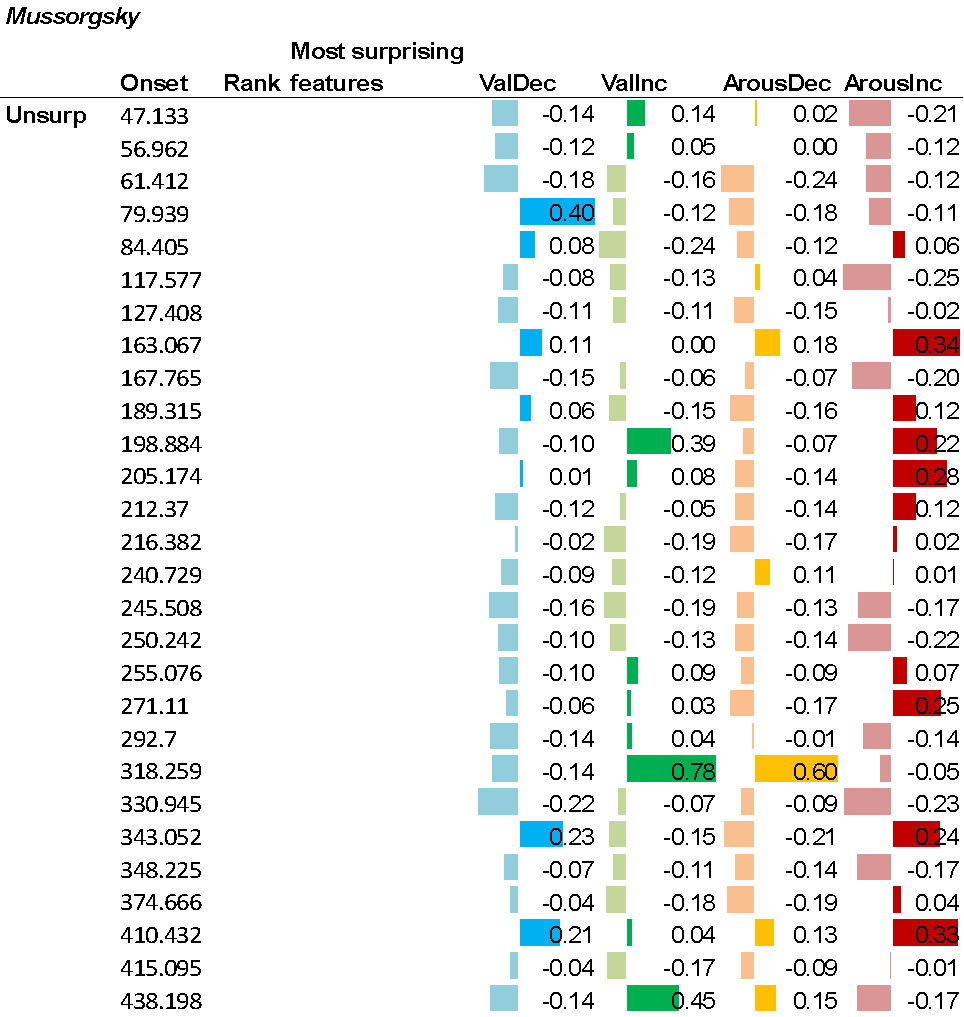
**Table S1.** **Cont.**


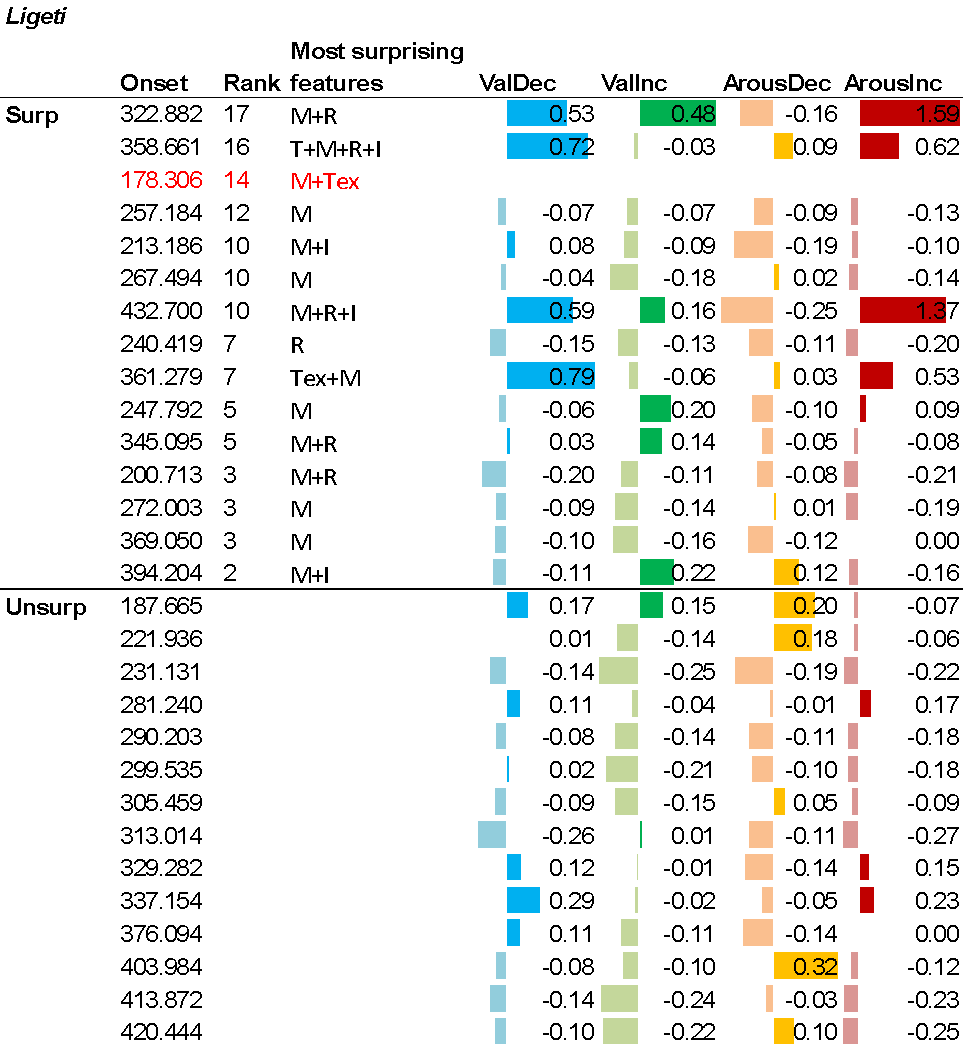


**Table S1.** **Cont. – non-analyzed piece**s


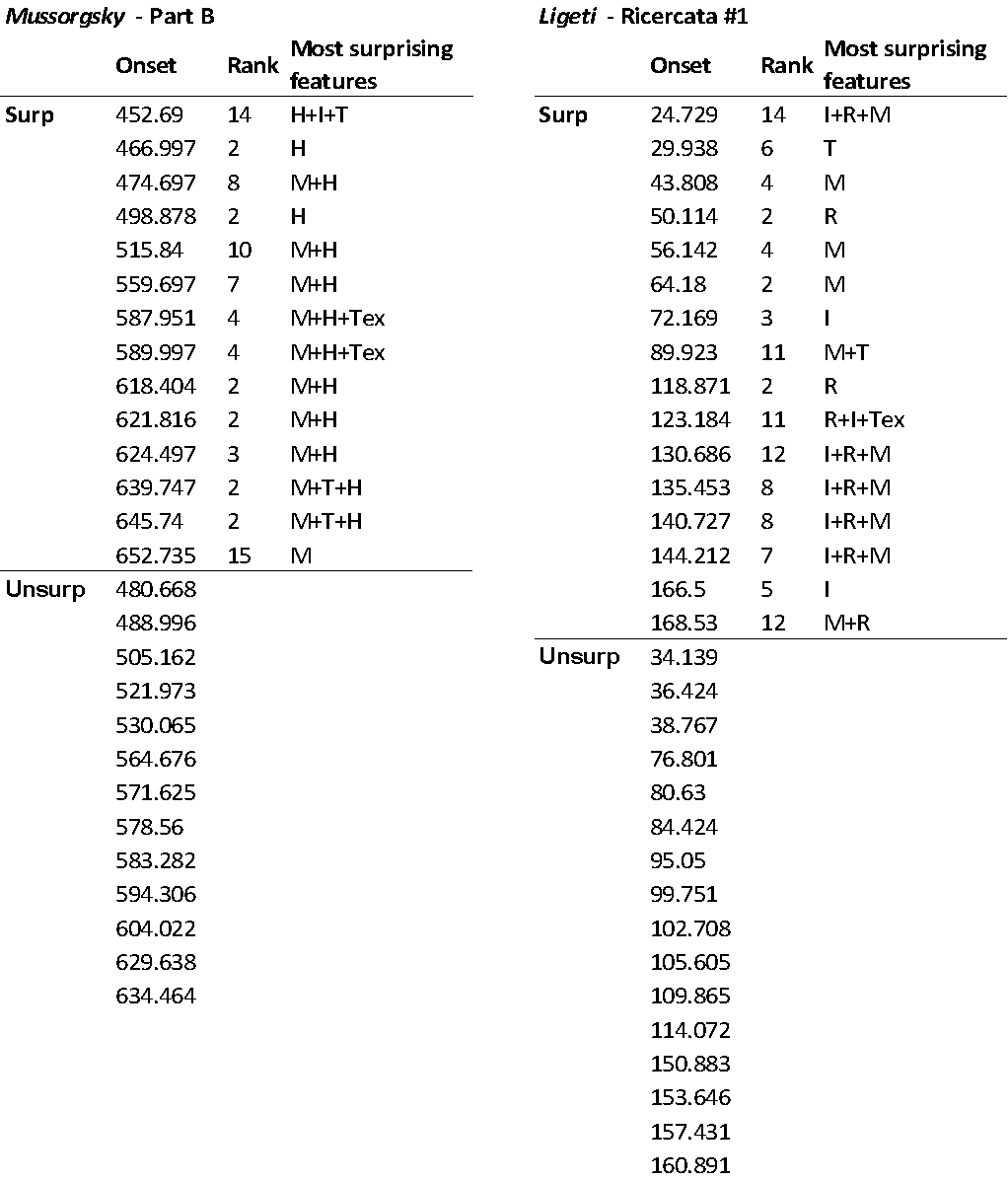


**Table S1. Details of experimental events.** In each row of the table, the following details are presented for each of the surprising and unsurprising events that were used in the behavioral and fMRI analyses: exact event onset in a millisecond resolution; the rank of the specific event, which represents the total number of judges who identified that event as surprising; the most surprising features of that event, which is a summary of the most agreed-upon musical features that arouse a sense of surprise among the musically-experienced judges, based on their verbal comments; and the mean of each of the affective ratings (z-score) that were computed for that event (valence decrease - blue, valence increase – green, arousal decrease – orange, arousal increase – red). Surprising events that were discarded from the behavioral and fMRI analysis due to: (a) less than a 1 TR (3 s) interval from other higher surprises, or (b) their occurrence right at the beginning of a musical piece, are marked in red. Abbreviations in the "most surprising features" column stand for: H=Harmony - refers to harmonic deviations, such as the presentation of unexpected chords; R = Rhythm – refers to changes in the periodicity of timing patterns of musical events, for example the onset of a note at an unexpected time or presentation of a novel meter; M = Melody – refers to changes in the melodic line or the exploited pitch range, such as an addition of voices, presentation of novel melodic material and recurrence of a musical motive; T=tempo – refers to alterations in the speed of music, i.e. gradual accelerations or decelerations; I = Intensity – refers to changes in loudness; Tex = Texture – refers to changes in the relations between different voices in a composition. It is important to note that rather than operating independently, these features typically interact in music. Moreover, the information provided about the most surprising features per event is rather coarse, as it is based on the most common verbal comments regarding the cause of each surprise. Hence, it is likely that additional features changed as well during the onset of surprise, but they do not appear in the table as judges did not explicitly mention these changes in their reports. Additional abbreviations stand for: surprise (surp.), valence decrease (valdec), valence increase (valinc), arousal decrease (arousdec), arousal increase (arousinc).

| **Table S2. Characteristics of high- vs low-pleasantness groups for each musical piece** | | | | | | | | | | | | | | | | | | | |  |
| --- | --- | --- | --- | --- | --- | --- | --- | --- | --- | --- | --- | --- | --- | --- | --- | --- | --- | --- | --- | --- |
| ***Piece: Glass*** | |  |  |  |  |  |  |  | |  | |  | |  | |  | |  | | |
| *The entire cohort* |  | |  |  |  |  | *The fMRI cohort* | |  | |  | |  | |  | |  | |  |  |
|  |  | |  |  |  |  |  | |  | |  | |  | |  | |  | |  |  |
| **Group  Variable** | **High-pleasantness** | | | **Low-pleasantness** | |  | **Group  Variable** | | **High-pleasantness** | | | | **Low-pleasantness** | | | |  | |  |  |
|  | ***N* or *M*** | | **1 *SD*** | ***N* or *M*** | **1 *SD*** | ***P* value** |  | | ***N* or *M*** | | **1 *SD*** | | ***N* or *M*** | | **1 *SD*** | | ***P* value** | |  |  |
| ***N*** | 18 | |  | 20 |  |  | ***N*** | | 16 | |  | | 12 | |  | |  | |  |  |
| **Gender** |  | |  |  |  | .49 | **Gender** | |  | |  | |  | |  | | .15 | |  |  |
| Male | 7 | |  | 10 |  |  | Male | | 5 | |  | | 7 | |  | |  | |  |  |
| Female | 11 | |  | 10 |  |  | Female | | 11 | |  | | 5 | |  | |  | |  |  |
| **Age** | 24.64 | | 3.24 | 26.70 | 3.54 | .07 | **Age** | | 24.84 | | 3.32 | | 27.25 | | 4.14 | | .10 | |  |  |
| **Musical experience** |  | |  |  |  | .56 | **Musical experience** | |  | |  | |  | |  | | .91 | |  |  |
| Non-musicians | 11 | |  | 14 |  |  | Non-musicians | | 9 | |  | | 7 | |  | |  | |  |  |
| Musicians  (professional training>5 yr.) | 7 | |  | 6 |  |  | Musicians  (professional training>5 yr.) | | 7 | |  | | 5 | |  | |  | |  |  |
| **Familiarity** | 1.78 | | 0.88 | 1.85 | 0.99 | .92 | **Familiarity** | | 1.81 | | 0.91 | | 2.00 | | 1.13 | | .75 | |  |  |
| **Liking of piece** | 4.39 | | 0.70 | 2.90 | 0.97 | **.000** | **Liking of piece** | | 4.38 | | 0.72 | | 2.92 | | 1.24 | | **.001** | |  |  |
| **GEMS subscales** |  | |  |  |  |  | **GEMS subscales** | |  | |  | |  | |  | |  | |  |  |
| Wonder | 2.55 | | 0.89 | 1.80 | 0.62 | **.006** | Wonder | | 2.66 | | 0.88 | | 1.89 | | 0.66 | | **.018** | |  |  |
| Transcendence | 2.53 | | 0.82 | 2.00 | 0.70 | **.04** | Transcendence | | 2.61 | | 0.83 | | 2.13 | | 0.77 | | .12 | |  |  |
| Tension | 1.33 | | 0.63 | 1.74 | 0.70 | .07 | Tension | | 1.29 | | 0.61 | | 1.65 | | 0.49 | | .10 | |  |  |
| Tenderness | 2.97 | | 0.93 | 2.17 | 0.81 | **.008** | Tenderness | | 3.13 | | 0.84 | | 2.45 | | 0.84 | | **.045** | |  |  |
| Sadness | 1.61 | | 0.80 | 2.15 | 1.16 | .11 | Sadness | | 1.67 | | 0.83 | | 2.31 | | 1.35 | | .17 | |  |  |
| Power | 2.10 | | 0.80 | 1.72 | 0.70 | .13 | Power | | 2.04 | | 0.59 | | 1.65 | | 0.64 | | .11 | |  |  |
| Peacefulness | 3.52 | | 0.71 | 2.64 | 0.81 | **.001** | Peacefulness | | 3.66 | | 0.61 | | 2.88 | | 0.83 | | **.008** | |  |  |
| Nostalgia | 3.04 | | 0.90 | 2.76 | 1.05 | .39 | Nostalgia | | 3.25 | | 0.71 | | 3.13 | | 1.16 | | .74 | |  |  |
| Joyful | 2.25 | | 0.72 | 1.91 | 0.71 | .15 | Joyful | | 2.27 | | 0.66 | | 1.76 | | 0.68 | | .06 | |  |  |

| ***Piece: Mussorgsky*** |  | | |  |  | | | |  | |  |  |  | | | | |  | | |  | | | |  | | | |  | | | |  | |  |
| --- | --- | --- | --- | --- | --- | --- | --- | --- | --- | --- | --- | --- | --- | --- | --- | --- | --- | --- | --- | --- | --- | --- | --- | --- | --- | --- | --- | --- | --- | --- | --- | --- | --- | --- | --- |
| *The entire cohort* |  | | |  |  | | | |  | |  | *The fMRI cohort* | | | |  | | |  | | | |  | | | |  | | | |  | | | |  |
|  |  | | |  |  | | | |  | |  |  | | | |  | | |  | | | |  | | | |  | | | |  | | | |  |
| **Group  Variable** | **High-pleasantness** | | | | **Low-pleasantness** | | | | | |  | **Group  Variable** | | | | **High-pleasantness** | | | | | | | **Low-pleasantness** | | | | | | | |  | | | |  |
|  | ***N* or *M*** | **1 *SD*** | | | ***N* or *M*** | | | **1 *SD*** | | | ***P* value** |  | | | | ***N* or *M*** | | | **1 *SD*** | | | | ***N* or *M*** | | | | **1 *SD*** | | | | ***p* value** | | | |  |
| ***N*** | 15 |  | | | 22 | | |  | | |  | ***N*** | | | | 12 | | |  | | | | 14 | | | |  | | | |  | | | |  |
| **Gender** |  |  | | |  | | |  | | | .64 | **Gender** | | | |  | | |  | | | |  | | | |  | | | | .67 | | | |  |
| Male | 8 |  | | | 10 | | |  | | |  | Male | | | | 5 | | |  | | | | 7 | | | |  | | | |  | | | |  |
| Female | 7 |  | | | 12 | | |  | | |  | Female | | | | 7 | | |  | | | | 7 | | | |  | | | |  | | | |  |
| **Age** | 25.13 | 4.29 | | | 25.59 | | | 3.32 | | | .72 | **Age** | | | | 25.58 | | | 4.60 | | | | 26.29 | | | | 3.22 | | | | .65 | | | |  |
| **Musical experience** |  |  | | |  | | |  | | | .33 | **Musical experience** | | | |  | | |  | | | |  | | | |  | | | | .38 | | | |  |
| Non-musicians | 8 |  | | | 16 | | |  | | |  | Non-musicians | | | | 6 | | |  | | | | 10 | | | |  | | | |  | | | |  |
| Musicians  (professional training>5 yr.) | 6 |  | | | 6 | | |  | | |  | Musicians  (professional training>5 yr.) | | | | 5 | | |  | | | | 4 | | | |  | | | |  | | | |  |
| **Familiarity** | 1.79 | 1.12 | | | 2.68 | | | 1.36 | | | **.04** | **Familiarity** | | | | 1.82 | | | 1.17 | | | | 3.14 | | | | 1.46 | | | | **.03** | | | |  |
| **Liking of piece** | 4.07 | 0.73 | | | 3.18 | | | 1.33 | | | **.05** | **Liking of piece** | | | | 3.91 | | | 0.70 | | | | 3.64 | | | | 1.34 | | | | .89 | | | |  |
| **GEMS subscales** |  |  | | |  | | |  | | |  | **GEMS subscales** | | | |  | | |  | | | |  | | | |  | | | |  | | | |  |
| Wonder | 2.21 | 0.65 | | | 2.11 | | | 0.87 | | | .71 | Wonder | | | | 2.20 | | | 0.71 | | | | 2.32 | | | | 1.00 | | | | .73 | | | |  |
| Transcendence | 2.18 | 0.46 | | | 2.29 | | | 0.88 | | | .63 | Transcendence | | | | 2.24 | | | 0.46 | | | | 2.51 | | | | 0.96 | | | | .37 | | | |  |
| Tension | 1.50 | 0.70 | | | 2.41 | | | 0.84 | | | **.002** | Tension | | | | 1.53 | | | 0.73 | | | | 2.27 | | | | 0.76 | | | | **.02** | | | |  |
| Tenderness | 2.14 | 0.94 | | | 1.90 | | | 0.87 | | | .43 | Tenderness | | | | 2.27 | | | 0.90 | | | | 1.97 | | | | 0.90 | | | | .41 | | | |  |
| Sadness | 1.36 | 0.44 | | | 1.62 | | | 0.69 | | | .17 | Sadness | | | | 1.45 | | | 0.45 | | | | 1.74 | | | | 0.73 | | | | .27 | | | |  |
| Power | 2.13 | 0.79 | | | 2.11 | | | 0.99 | | | .95 | Power | | | | 2.15 | | | 0.83 | | | | 2.20 | | | | 1.02 | | | | .89 | | | |  |
| Peacefulness | 2.53 | 0.85 | | | 2.34 | | | 0.99 | | | .55 | Peacefulness | | | | 2.62 | | | 0.77 | | | | 2.21 | | | | 0.86 | | | | .23 | | | |  |
| Nostalgia | 2.32 | 0.61 | | | 2.28 | | | 0.91 | | | .89 | Nostalgia | | | | 2.43 | | | 0.63 | | | | 2.39 | | | | 0.88 | | | | .90 | | | |  |
| Joyful | 2.61 | 0.77 | | | 2.16 | | | 0.64 | | | .07 | Joyful | | | | 2.68 | | | 0.80 | | | | 2.24 | | | | 0.70 | | | | .15 | | | |  |
| ***Piece: Ligeti*** |  | |  | | |  |  | | |  | |  | |  | | |  | | | | |  | | | |  | | | |  | | | |  | |
| *The entire cohort* |  | |  | | |  |  | | |  | | *The fMRI cohort* | | |  | | | | |  | | | |  | | | |  | | | |  | | |  |
|  |  | |  | | |  |  | | |  | |  | | |  | | | | |  | | | |  | | | |  | | | |  | | |  |
| **Group  Variable** | **High-pleasantness** | | | | | **Low-pleasantness** | | | |  | | **Group  Variable** | | | **High-pleasantness** | | | | | | | | | **Low-pleasantness** | | | | | | | |  | | |  |
|  | ***N* or *M*** | | **1 *SD*** | | | ***N* or *M*** | **1 *SD*** | | | ***p* value** | |  | | | ***N* or *M*** | | | | | **1 *SD*** | | | | ***N* or *M*** | | | | **1 *SD*** | | | | ***p* value** | | |  |
| ***N*** | 18 | |  | | | 21 |  | | |  | | ***N*** | | | 15 | | | | |  | | | | 15 | | | |  | | | |  | | |  |
| **Gender** |  | |  | | |  |  | | | .40 | | **Gender** | | |  | | | | |  | | | |  | | | |  | | | | .14 | | |  |
| Male | 7 | |  | | | 11 |  | | |  | | Male | | | 5 | | | | |  | | | | 9 | | | |  | | | |  | | |  |
| Female | 11 | |  | | | 10 |  | | |  | | Female | | | 10 | | | | |  | | | | 6 | | | |  | | | |  | | |  |
| **Age** | 1.74 | | 0.57 | | | 1.67 | 0.60 | | | .13 | | **Age** | | | 25.13 | | | | | 3.60 | | | | 27.03 | | | | 3.57 | | | | .16 | | |  |
| **Musical experience** |  | |  | | |  |  | | | .17 | | **Musical experience** | | |  | | | | |  | | | |  | | | |  | | | | .44 | | |  |
| Non-musicians | 10 | |  | | | 16 |  | | |  | | Non-musicians | | | 9 | | | | |  | | | | 11 | | | |  | | | |  | | |  |
| Musicians  (professional training>5 yr.) | 8 | |  | | | 5 |  | | |  | | Musicians  (professional training>5 yr.) | | | 6 | | | | |  | | | | 4 | | | |  | | | |  | | |  |
| **Familiarity** | 1.83 | | 1.25 | | | 2.24 | 1.41 | | | .44 | | **Familiarity** | | | 1.93 | | | | | 1.33 | | | | 1.93 | | | | 1.22 | | | | .97 | | |  |
| **Liking of piece** | 2.56 | | 0.98 | | | 1.71 | 0.72 | | | **.008** | | **Liking of piece** | | | 2.47 | | | | | 1.06 | | | | 1.80 | | | | 0.68 | | | | .07 | | |  |
| **GEMS subscales** |  | |  | | |  |  | | |  | | **GEMS subscales** | | |  | | | | |  | | | |  | | | |  | | | |  | | |  |
| Wonder | 1.74 | | 0.57 | | | 1.67 | 0.60 | | | .69 | | Wonder | | | 1.69 | | | | | 0.61 | | | | 1.79 | | | | 0.62 | | | | .66 | | |  |
| Transcendence | 1.74 | | 0.61 | | | 1.78 | 0.47 | | | .83 | | Transcendence | | | 1.77 | | | | | 0.64 | | | | 1.88 | | | | 0.47 | | | | .59 | | |  |
| Tension | 2.54 | | 0.80 | | | 3.64 | 0.77 | | | **.000** | | Tension | | | 2.52 | | | | | 0.84 | | | | 3.72 | | | | 0.72 | | | | **.000** | | |  |
| Tenderness | 1.50 | | 0.46 | | | 1.20 | 0.31 | | | **.03** | | Tenderness | | | 1.49 | | | | | 0.48 | | | | 1.24 | | | | 0.35 | | | | .11 | | |  |
| Sadness | 1.48 | | 0.38 | | | 1.83 | 0.77 | | | .08 | | Sadness | | | 1.49 | | | | | 0.42 | | | | 1.78 | | | | 0.80 | | | | .23 | | |  |
| Power | 1.50 | | 0.36 | | | 1.55 | 0.54 | | | .72 | | Power | | | 1.53 | | | | | 0.38 | | | | 1.61 | | | | 0.58 | | | | .66 | | |  |
| Peacefulness | 2.04 | | 0.60 | | | 1.41 | 0.42 | | | **.000** | | Peacefulness | | | 2.12 | | | | | 0.58 | | | | 1.48 | | | | 0.43 | | | | **.002** | | |  |
| Nostalgia | 1.99 | | 0.67 | | | 1.76 | 0.62 | | | .28 | | Nostalgia | | | 2.03 | | | | | 0.68 | | | | 1.82 | | | | 0.70 | | | | .40 | | |  |
| Joyful | 1.92 | | 0.59 | | | 2.00 | 0.58 | | | .66 | | Joyful | | | 1.92 | | | | | 0.65 | | | | 2.06 | | | | 0.58 | | | | .56 | | |  |

**Table S2. Characteristics of high- vs low-pleasantness groups for each musical piece.** Demographic characteristics of both subgroups, as well as information regarding their musical expertise, familiarity with the musical stimuli and subjective ratings of the emotional experience (liking, subscales of the Geneva Emotional Musical Scales (GEMS) questionnaire) are presented for each musical piece. Details are provided for the entire study cohort (white background), as well as separately for participants that were included in the fMRI analyses (grey background). Differences between groups were estimated using an independent two-sample t-test for age of participants; a z-test for comparing two population proportions for measures of familiarity and liking, as well as for subscales of the GEMS questionnaire; and a Chi square test for goodness of fit for assessing differences in frequency of expert musicians and gender identity between groups. *P*-values of comparisons are denoted in the right column, and significant differences (*p*<.05, uncorrected) are emphasized in black.

**Supplemental Methods**

***Participants.*** The participants had no known history of neurological or psychiatric disorders, and provided written informed consent according to the Tel-Aviv Sourasky Medical Center institutional review board (IRB) committee guidelines prior to the experiment. Thirteen of the participants had more than five years of experience playing music (Chapin et al., 2010), ranging between 7 and 22 years (Mexperience=12.31±4.75 years). Musical experience of the remaining 27 participants ranged between 0 and 5 years (Mexperience=1.94±1.67 years).

***Affective Features of Musical Stimuli.*** The three musical pieces were shown in a pre-test (n=17) to elicit clear yet quantitatively different affective experiences in terms of their averaged continuous valence, which was relatively positive in Glass (*M*=.25±.31), neither positive nor negative in Mussorgksy (*M*=.02±.29) and negative in Ligeti (*M*=-.43±.30) (Glass vs. Ligeti: *Z*=3.07, *p*=.002; Mussorgsky vs. Ligeti: *Z*=2.39, *p*=.016; Glass vs. Mussorgsky: *Z*=1.47, *p*=.14). Averaged continuous arousal was slightly lower in Glass (*M*=.07±.23) than in Mussorgksy (*M*=.16±.23) and Ligeti (*M*=.12±.31). Pairwise comparisons of averaged arousal did not reach significance in the pretest (Glass vs. Ligeti: *Z*=1.21, *p*=.22; Mussorgsky vs. Ligeti: *Z*=0.00, *p*=1.0; Glass vs. Mussorgsky: *Z*=1.47, *p*=.14). Yet, both patterns of differences between pieces in valence (i.e. Glass>Mussorgsky>Ligeti) and arousal (i.e. Mussorgsky & Ligeti>Glass) were confirmed and reached significance in the main experiment (see Supplemental Results).

***Characteristics of Musical Stimuli.*** We used three prolonged pieces of western art music in this study, composed by Phillip Glass, Modest Mussorgsky and György Ligeti. . These pieces were selected due to their capability to elicit a range of dynamic emotional experiences (based on the above-described pre-test) as well as musical surprises.

Philip Glass's *The Hours* is a late 20^th^ century piece of light film music from the soundtrack of the film *The Hours.* It is clearly based on harmonic progressions creating a clear harmonic rhythm contrasting major and minor sections, which resembles the harmonic structure that can be found in popular music. It is highly structured with exact and varied repetitions of phrases or whole sections. In addition, it presents contrasts between successive or simultaneous duple and triple meter.

Ligeti's Ricercatas, composed during the middle 20^th^ century, are a series of 11 piano pieces exploring different compositional possibilities using an increasing number of pitch classes ranging from only two (A and D as the final tone) in the first Ricercata, three (E#, F#, G#) in the second Ricercata (used in the current analysis) and up to 12 pitch classes in the final 11th piece. The second Ricercata is very ordered in the temporal domain, using contrasts of register, loudness, texture and consonance versus dissonance. Nonetheless, it displays a certain degree of temporal irregularity due to the long periods of silence in between its phrases.

*Night on Bald Mountain* is a Russian piece from the late 19^th^ century which includes numerous Russian folk motives, chromatic inflections, syncopated rhythms and instances of dissonant colorations. Part A (0:00-7:32) of the piece is characterized by a fast Allegro feroce tempo, varied rhythms and a dense texture. Part B (7:33-10:57) of the piece is a slower and gentle section, uniform in rhythm and light in texture.

The three selected pieces were apt for testing responses to musical surprises for a number of reasons. While differing on many dimensions, the Glass and Ligeti pieces are both relatively simple to model in terms of pitch content or harmonic structure. Given the relative simplicity of these pieces, we expected that surprises could be recognized in them quite clearly. In contrast, Mussorgsky's *Night on Bald Mountain*, a Russian piece from the late 19th century, is much more complex than Glass and Ligeti due to its exploitation of rich dynamics, large contrasts between pitch ranges and syncopated rhythms. While this music may be more "noisy" and less easy to follow, we used it since an excerpt from it was rated as fearful and as highly surprising in previous studies (e.g., Krumhansl, 1997).

The Glass and Ligeti pieces were recorded using a Yamaha Disklavier upright piano. This is an acoustic piano with a MIDI output, allowing the storage of the exact details of each keystroke (e.g., pitch and velocity). The recorded sound files were passed through a music compression procedure using a built-in multiband compressor of Cubase 5 software (Steinberg Media Technologies, Germany). Recordings of the Glass's and Ligeti's files are attached as supplementary sound files to Singer *et al.*, 2016. For Mussorgsky's piece, we used an audio recording of *Night on Bald Mountain* performed by Boris Berezovsky, from the album [*Piano Works of Russia*](http://www.allmusic.com/album/release/piano-works-of-russia-mr0002719622) (Teldec, 1996).

***Annotation and Ranking of Musical Surprises***. The surprise annotation procedure took place during three consecutive listening sessions. In the first session the judges were asked to tap to the beat. In the second session they marked online any musical events that sounded surprising to them via the interface of the Sonic Visualizer software (version 1.7.2; Cannam *et al.*, 2010). In the third session they commented verbally about what exactly was surprising in each event, and had a chance to correct the timing of each marking. The most surprising features reported by the judges per surprise are summarized in Table S1.

Surprise annotations were initially depicted per second, thus yielding a time series denoting how surprising each second "collectively" felt on a scale potentially ranging between 0 and 20 for each piece. As there were slight differences in marking the onset timings across participant, surprise rankings referring to the same event were sometimes scattered across a few seconds (1-4). This time series served as a basis for extracting surprising events for the analysis, whose timings were aligned to the precise onset of the musical event that triggered them. This was achieved by author O.S., who matched judges' verbal reports regarding the features of each surprise together with the musical events depicted in the scores.

Inclusion criteria of surprises were as follows: a) events in which the unfolding of surprising musical material lasted up to 1s, as these were most suitable for the fMRI design. Consequently, surprising musical events that unfolded over longer durations, such as ritartandos (i.e. a gradual decrease in tempo) were excluded from further analysis; b) events ranked as surprising by more than one judge; c) events occurring at least 21s (equal to 7 TRs) after the beginning of a musical piece, or right at the beginning of new musical subsections (i.e. Ligeti's 2^nd^ Ricercata and part B of Mussorgsky's piece). This criteria was defined in order to avoid both the non-reliable rating responses (Schubert, 2013), and the exceptionally strong subcortical brain activity (Mueller *et al.*, 2015) which are associated with the beginning of music presentation. Note that surprises from two musical sections were not analyzed: part B of Mussorgsky's piece, because a rather small set of surprises characterized by low rankings was found in this section (9 of the 14 surprises were ranked 4 or lower); and Ricercata no. 1 by Ligeti, due to its short duration and the fact that a relatively large portion of the surprises (50%) consisted of silent events and prolonged musical events.

We additionally tested whether experimental conditions differed in terms of low-level acoustical features associated with auditory cortex activation (Alluri *et al.*, 2012; Lehne *et al.*, 2013; Trost *et al.*, 2015). To this end, we used the MIR toolbox (Lartillot and Toiviainen, 2007) to extract values of information related to pitch (specifically chromagram – which indicates the dispersion of sound energy over the 12 pitch classes), loudness (RMS - the root mean square of sounds' global energy) and event density. Subsequently, we conducted nonparametric independent pairwise comparisons (Kruskal-Wallis test) between all pairs of conditions in each piece with regards to each feature. No significant differences were found in either Glass (all *p*s>.16), Mussorgsky (all *p*s>.19) or Ligeti (all *p*s>.43). In addition, results of a multiple regression analysis we performed in order to test the contribution of *changes* in several musical features to the musical surprises in each piece are presented in the upcoming Supplemental Results section.

***Acquisition of Continuous Affect Ratings.*** Participants indicated their real-time subjective feeling using the Emujoy software (Nagel *et al.*, 2007) on a two-dimensional emotion response space that encompassed valence (i.e. pleasantness, horizontal axis) and arousal (i.e. activation, vertical axis) by moving a computer's mouse cursor. Each cursor movement was recorded by the software at a maximal rate of 20 Hz. The participants were explicitly instructed to indicate their own felt (and not perceived) emotions and to relate as they can to the experience they had during scanning. The participants were given an opportunity to practice the use of the software and to familiarize with the concepts of valence and arousal in a separate practice session.

***Rating Session of Mussorgsky's Piece in the Scanner.*** Twenty-three of the participants heard Mussorgsky's piece twice in the scanner, meaning that in total these participants heard this piece three times and rated it twice. This rating session immediately followed the passive listening session. The continuous rating method varied between these participants as follows: out of the first 11 of these participants, 10 rated their felt level of arousal and 1 rated her felt level of valence – both on a unidimensional axis by using an in-house software (Raz et al., 2012). The remaining 12 participants rated their felt valence and arousal on a two-dimensional axis via the Emujoy software (Nagel *et al.*, 2007) by moving an MRI-compatible joystick. Collection of these data was unreliable due to technical issues, and thus it was not analyzed. Moreover, the number of participants who listened to Mussorgsky twice in the scanner was similar between the pleasantness-based subgroups we identified: 10/15 in the high-pleasantness group and 12/22 in the low-pleasantness group for the entire cohort on which we based the behavioral analysis (*χ*²(1)=.54, *p*=.46); and 8/12 in the high-pleasantness group and 9/14 in the low-pleasantness group for the fMRI cohort on which we based the ROI analysis (*χ*²(1)=.016, *p*=.89)**.** In addition, the level of familiarity with Mussorgsky's piece (which was rated during the behavioral session after all the musical stimuli were presented) did not differ between participants who heard the piece twice in the scanner and those who did not (*Z*=-.05, *p*=.96 for the entire cohort; and *Z*=.84, *p*=.39 for the fMRI cohort).

***fMRI acquisition and data preprocessing***

***MRI Data Acquisition.*** Structural and functional scans were performed using a GE 3 T Signa Excite echo speed scanner with an 8-channel head coil. Functional whole-brain scans were performed in an interleaved top-to-bottom order, using a T2*-weighted gradient-echo echo-planar imaging sequence (TR/TE = 3000/35 ms, flip angle = 90°, 128 × 128 matrix, FOV = 220 × 220 mm, 39 slices per volume with 3 mm thickness and no gap). Functional images of two participants were acquired using 38 slices per volume due to a technical limitation. A total of 184 volumes were acquired for the Glass session, 262 volumes for the Mussorgksy session and 200 for the Ligeti session. Subsequent to the functional scanning, a T1-weighted 3D axial spoiled gradient echo (SPGR) pulse sequence (TR/TE = 8.9/3.5 ms, flip angle = 13°, voxel size = 1 ∗ 1 ∗ 1 mm, FOV=256 × 256 mm, slice thickness=1mm) was applied to provide high-resolution structural images.

***fMRI Preprocessing.*** The data were preprocessed using BrainVoyager QX version 2.3 software (Brain Innovation, Maastricht, The Netherlands) and in house software developed in Matlab. The first 10 volumes of the acquisition were discarded to allow for stabilization of the magnetic field. Slice scan time correction was performed using sinc interpolation. Head motion correction was performed by spatially aligning all volumes to the middle volume via rigid body transformations using sinc interpolation. Linear trend removal and temporal high-pass filtering were applied to each voxel's time-course to remove linear and nonlinear low-frequency drifts of 3 or fewer cycles per time course (high pass filter of 0.005 Hz). A spatial smoothing with a 6 mm FWHM Gaussian Kernel was used. The structural and functional images were manually co-registered and transformed into the same Talairach space. Criteria for discarding fMRI datasets in the current study included head movements that exceeded 2mm and a presence of more than one spike larger than 1mm in the head movement regressors.

**Supplemental Results**

***Testing the contribution of different musical features to musical surprise.*** In order to estimate the contribution of different musical features to the surprise model, we conducted a stepwise multiple regression in each piece with the SPSS 20 software (IBM, Armonk, New York)***.*** We used the MIR (Lartillot and Toiviainen, 2007) and PsySound 3 (Cabrera *et al.*, 2007) toolboxes to extract several musical and acoustic features that capture: a) fluctuations in rhythmic aspects (pulse clarity [i.e. beat strength], tempo and event density), b) timbre and spectral content (brightness and spectral centroid; these measures describe the centering of sound energy around high- or low-frequency registers throughout music), c) pitch-related information (Chromagram; indicates the dispersion of sound energy over the 12 pitch classes), d) level of sensory dissonance (roughness), and e) loudness (RMS [i.e. the root mean square of sounds' global energy] and dynamic loudness; the latter feature, which captures the subjective impression of sound intensity, is the only feature we extracted with PsySound 3). MIDI files that were obtained from the Disklavier recordings of the Glass and Ligeti pieces allowed the addition of two relevant features to the regression models performed for these pieces – the number of notes (akin to event density) and mean pitch. Selection of musical features was based on previous fMRI studies of musical feature analysis (Alluri *et al.*, 2012; Trost *et al.*, 2015). All musical features were down-sampled to 1Hz. Since we intended to associate the strength of momentary *changes* in these features with surprise level, we first calculated a time series for each feature wherein the value in each second was defined as the absolute value of a subtraction between the value at the current second and the value at the previous second (i.e., absolute value of the first derivative). Next, we extracted the *change* values occurring at the precise second of all surprising and unsurprising events for each feature, and these values served as independent predictors in the model. The surprise level, defined as the number of judges who ranked a certain event as surprising (unsurprising events were ranked 0), was the dependent variable.

The multiple regression model was significant in all three pieces (Glass: *R*²=.40, Adjusted *R*²=.37, *F*(3,47)=10.6, *p*<.001; Mussorgsky: *R*²=.13 , Adjusted *R*²=.11, *F*(2,81)=5.87, *p*<.005; Ligeti: *R*²=.21 , Adjusted *R*²=.18, *F*(1,26)=6.72, *p*=.015). The surprise model in each piece was predicted by different musical features, as follows: In Glass, variance of surprise level was significantly explained by greater difference in RMS (*β*=.393, *t*=3.03, *p*<.005, *95%CI*: [0.132, 0.653]) and roughness (*β*=.262, *t*=2.02, *p*<.05, *95%CI*: [0.001, 0.522]), but also by reduced differences in mean pitch (*β*=-.281, *t*=-2.49, *p*=.016, *95%CI*: [-0.507, -0.054]). In Mussorgksy, higher difference in spectral centroid (*β*=.231, *t*=2.21, *p*<.05, *95%CI*: [.023, .438]) and chromagram (*β*=.249, *t*=2.39, *p*<.05, *95%CI*: [.041, .457]) contributed significantly to the surprise model. In Ligeti, only chromagram differences predicted surprise level (*β*=.453 *t*=2.59, *p*=.015, *95%CI*: [.094, .813]).

***Total Valence and Arousal of the Three Musical Pieces.*** To account for differences in total valence and arousal experienced in response to each musical piece, we calculated the grand average of the continuous arousal and valence ratings. Ratings of valence corroborated the preliminary results from the pretest, as Glass's piece was experienced as more pleasant than the Mussorgsky (*t*(34)=2.55, *p*=.005) and Ligeti (*t*(36)=9.16, *p*<.001) pieces, and Mussorgsky's music was more pleasant compared to Ligeti (*t*(35)=9.44, *p*<.001). In terms of arousal, the Glass piece was experienced as less arousing relative to both Mussorgsky (*t*(34)=-3.06, *p*<.005) and Ligeti (*t*(36)=-3.47, *p*<.005). Arousal did not differ significantly between Mussorgsky and Ligeti (*p*>.24).

***Classification of Subgroups Experiencing High versus Low Levels of Pleasantness in Response to the Music.*** The final cluster centers of the valence ratings for the high- and low-pleasantness groups were as follows: In Glass *M*=0.643 for the high-pleasantness group and *M*=0.138 for the low-pleasantness group; In Ligeti, *M*=0.034 for the high-pleasantness group and *M*=-0.452 for the low-pleasantness group; and in Mussorgsky, *M*=0.476 for the high-pleasantness group and *M*=-0.002 for the low-pleasantness group.

***Interaction of Pleasantness and Surprise Level in the NAcc in Mussorgsky's Piece While Controlling for Familiarity.*** As familiarity differed between the high- and low-pleasantness groups, we ran an additional repeated-measures ANOVA with familiarity level as a covariate. The group x surprise interaction remained significant (*F*(1,46)=3.97, *p*=.026, ηp*^2^*=.15), and familiarity x surprise interaction was nonsignificant (*F*(1,46)=1.00, *p*=.37, ηp*^2^*=.04). Significance of pairwise comparisons was weakened in the high-pleasantness group (HS vs. US: *F*(1,23)=2.55, *p*=.12; HS vs. LS*: F*(1,23)=4.36, *p*=.048). However, significance level of the LS vs. US contrast in the low-pleasantness group was strengthened *F*(1,23)=6.82, *p*=.016). The between-groups effect for HS was reduced as well (*F*(1,23)=4.79, *p*=.038).

**Supplemental References**

Alluri, V., Toiviainen, P., Jääskeläinen, I.P., et al. (2012). Large-scale brain networks emerge from dynamic processing of musical timbre, key and rhythm. *NeuroImage*, **59**, 3677–89.

Cabrera, D., Ferguson, S., Schubert, E. (2007). ‘Psysound3’: Software for Acoustical and Psychoacoustical Analysis of Sound Recordings. In: Georgia Institute of Technology.

Cannam, C., Landone, C., Sandler, M. (2010). Sonic visualiser: An open source application for viewing, analysing, and annotating music audio files. In: *Proceedings of the 18th ACM international conference on Multimedia*. ACM, p. 1467–1468.

Koelsch, S. (2014). Brain correlates of music-evoked emotions. *Nature Reviews Neuroscience*, **15**, 170–180.

Krumhansl, C.L. (1997). An exploratory study of musical emotions and psychophysiology. *Canadian Journal of Experimental Psychology/Revue canadienne de psychologie expérimentale*, **51**, 336–53.

Lartillot, O., Toiviainen, P. (2007). A Matlab toolbox for musical feature extraction from audio. In: *International conference on digital audio effects*. Bordeaux, FR, p. 237–244.

Lehne, M., Rohrmeier, M., Koelsch, S. (2013). Tension-related activity in the orbitofrontal cortex and amygdala: an fMRI study with music. *Social Cognitive and Affective Neuroscience*, **9**, 1515–1523.

Mueller, K., Fritz, T., Mildner, T., et al. (2015). Investigating the dynamics of the brain response to music: A central role of the ventral striatum/nucleus accumbens. *NeuroImage*, **116**, 68–79.

Nagel, F., Kopiez, R., Grewe, O., et al. (2007). EMuJoy: Software for continuous measurement of perceived emotions in music. *Behavior Research Methods*, **39**, 283–290.

Raz, G., Winetraub, Y., Jacob, Y., et al. (2012). Portraying emotions at their unfolding: a multilayered approach for probing dynamics of neural networks. *Neuroimage*, **60**, 1448–1461.

Schubert, E. (2013). Reliability issues regarding the beginning, middle and end of continuous emotion ratings to music. *Psychology of Music*, **41**, 350–71.

Singer, N., Jacoby, N., Lin, T., et al. (2016). Common modulation of limbic network activation underlies musical emotions as they unfold. *NeuroImage*, **141**, 517–529.

Trost, W., Frühholz, S., Cochrane, T., et al. (2015). Temporal dynamics of musical emotions examined through intersubject synchrony of brain activity. *Social cognitive and affective neuroscience*, **10**, 1705-21.
